# Prognostic models for predicting postoperative recurrence in Crohn’s disease: a systematic review and critical appraisal

**DOI:** 10.3389/fimmu.2023.1215116

**Published:** 2023-06-30

**Authors:** Rirong Chen, Jieqi Zheng, Chao Li, Qia Chen, Zhirong Zeng, Li Li, Minhu Chen, Shenghong Zhang

**Affiliations:** ^1^ Department of Gastroenterology, The First Affiliated Hospital, Sun Yat-sen University, Guangzhou, China; ^2^ Department of Clinical Medicine, Zhongshan School of Medicine, Sun Yat-Sen University, Guangzhou, China

**Keywords:** Crohn’s disease, postoperative recurrence, prognostic model, patient management, systematic review

## Abstract

**Background and Aims:**

Prophylaxis of postoperative recurrence is an intractable problem for clinicians and patients with Crohn’s disease. Prognostic models are effective tools for patient stratification and personalised management. This systematic review aimed to provide an overview and critically appraise the existing models for predicting postoperative recurrence of Crohn’s disease.

**Methods:**

Systematic retrieval was performed using PubMed and Web of Science in January 2022. Original articles on prognostic models for predicting postoperative recurrence of Crohn’s disease were included in the analysis. The risk of bias was assessed using the Prediction Model Risk of Bias Assessment (PROBAST) tool. This study was registered with the International Prospective Register of Systematic Reviews (PROSPERO; number CRD42022311737).

**Results:**

In total, 1948 articles were screened, of which 15 were ultimately considered. Twelve studies developed 15 new prognostic models for Crohn’s disease and the other three validated the performance of three existing models. Seven models utilised regression algorithms, six utilised scoring indices, and five utilised machine learning. The area under the receiver operating characteristic curve of the models ranged from 0.51 to 0.97. Six models showed good discrimination, with an area under the receiver operating characteristic curve of >0.80. All models were determined to have a high risk of bias in modelling or analysis, while they were at low risk of applicability concerns.

**Conclusions:**

Prognostic models have great potential for facilitating the assessment of postoperative recurrence risk in patients with Crohn’s disease. Existing prognostic models require further validation regarding their reliability and applicability.

**Systematic review registration:**

https://www.crd.york.ac.uk/PROSPERO/, identifier CRD42022311737.

## 1 Introduction

Crohn’s disease (CD), characterised by the presence of lesions and transmural inflammation, is a chronic relapsing inflammatory bowel disease capable of causing irreversible and disabling damage (1). Owing to disease damage, approximately half of all patients with CD need surgery within 10 years of diagnosis (2). However, surgery is not curative. It has been reported that 35%–85% of patients experience endoscopic recurrence within one year, and approximately half undergo reoperation within 10 years (3, 4). Prophylaxis of postoperative recurrence of CD remains a challenge for both clinicians and researchers. A randomised clinical trial revealed that treatment strategies that consider the risk of recurrence may be effectively used to reduce postoperative recurrence rates after CD-related surgery. This finding underscores the importance of stratifying patients with CD based on their recurrence risk (5). Therefore, the prediction of postoperative recurrence has received considerable attention, facilitating the personalised management of patients with postoperative CD.

Many risk factors for the postoperative recurrence of CD have been established, including smoking, previous resection, early disease onset, short disease duration, perianal disease, penetrating behaviour, and extensive bowel disease (3, 6). Nevertheless, no definitive means for stratifying the risk of postoperative recurrence in patients with CD has been established, and improved tools for identifying at-risk patients are urgently needed. Prognostic models, mathematical equations that combine multiple variables to estimate the probability of a specific endpoint, have been increasingly used. Since prognostic models comprehensively consider prognostic information due to the presence of various risk factors, they have the potential to improve predictive accuracy (7). Thus, several prognostic models for predicting postoperative recurrence in CD have been developed, and their utility for risk stratification and guiding adjuvant treatment decisions have been discussed.

This study aimed to systematically review the literature to identify models for predicting postoperative recurrence in CD and assess model performance. In addition, we critically appraised prediction models to evaluate their risk of bias (ROB) and applicability.

## 2 Methods

The conduction and reporting of this systematic review adhered to the Preferred Reporting Items for Systematic Reviews and Meta-Analyses (PRISMA) statement (8). The protocol was registered in the International Prospective Register of Systematic Reviews (PROSPERO), and the registration number is CRD42022311737.

### 2.1 Eligibility criteria and search strategy

We included original articles that developed or validated a score or model for predicting postoperative recurrence in patients who underwent CD-related intestinal surgery. Cohort and case-control studies were also included. Studies in languages other than English, those without full text, or those with insufficient data for assessing model performance were excluded.

We searched PubMed and Web of Science, with the last search performed on January 25, 2022. Search terms used included Crohn’s disease, surgery or postoperative, recurrence or relapse, and their respective synonyms. Prognostic models were retrieved with reference to a search filter updated by Geersing et al. (9), plus other keywords with wildcards, including stratification, receiver-operating characteristic curve or ROC curve, discrimination, calibration, c-statistic, area under the curve or AUC, indices, algorithm, and multivariable. Detailed search strategies are provided in **Supplementary Table 1**.

### 2.2 Study selection and data extraction

After reaching a consensus regarding eligibility criteria, two reviewers (JZ and QC) independently initially screened the titles and abstracts of all retrieved articles. Thereafter, the researchers screened the full text of selected articles to determine which would be included in the final analysis. If any inconsistencies were encountered, a consensus was obtained via consultation. If necessary, a third reviewer (RC) was consulted to reach a final decision.

Data were independently extracted by two reviewers (JZ and CL). Disagreements were resolved by reaching a consensus. Under the guidance of the Checklist for critical appraisal and data extraction for systematic reviews of prediction modelling studies (CHARMS) (10), the following data were extracted: primary author, year of publication, the country in which the study was conducted, study design and source of data, participant characteristics, number and type of candidate predictors, outcomes and time horizon, sample size, missing data and its handling, modelling method, model presentation, model performance, and validation method.

### 2.3 Model performance and risk of bias assessment

Calibration and discrimination were used to quantify prognostic model performance. Model calibration was evaluated using calibration-in-the-large and calibration slope. Discrimination was assessed using the area under the curve (AUC) of the receiver operating characteristic (ROC) or coincidence statistic. The sensitivity and specificity with corresponding cut-off values served as auxiliary to evaluate model performance if the above measures were unavailable.

To assess the ROB and applicability of each study, the Prediction Model Risk of Bias Assessment (PROBAST) tool was adapted (11). The tool consists of four domains (participants, predictors, outcome, and analysis) for ROB assessment and three domains (participants, predictors, and outcome) for the evaluation of applicability concerns. ROB and applicability were rated as low, high, or unclear for each domain. Only if all domains were rated as having low ROB can a study be considered to have an overall low ROB, and this rule also applies to applicability ratings. Two reviewers participated in this process, with one reviewer (JZ) performing the assessment and the other reviewer (RC) conducting the inspection. Any disagreements were resolved by consensus. A detailed assessment of each study is provided in **Supplementary Table 2**.

### 2.4 Data synthesis

Results were summarised via tabulation and narrative synthesis. Quantitative synthesis of prognostic models was not feasible in this systematic review due to the heterogeneity of studies.

## 3 Results

**Figure 1:**
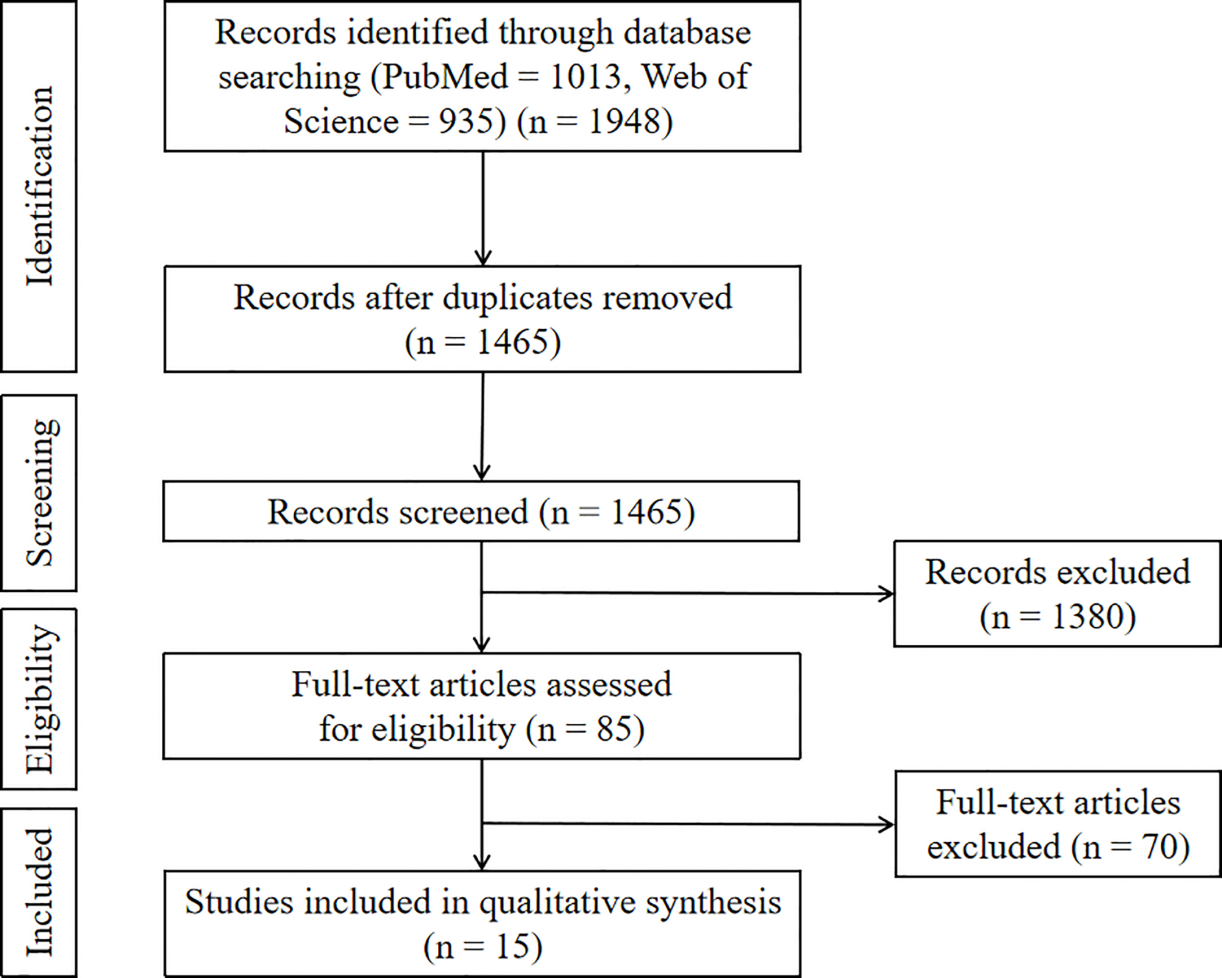
Flow chart.

We retrieved 1948 articles from PubMed and Web of Science databases, 1465 of which remained after removing duplicate articles. Through title and abstract screening, 1380 studies that failed to meet the eligibility criteria were removed. Thereafter, the remaining 85 studies were subjected to full-text screening. Seventy studies were excluded because they focused only on prognostic factors without modelling, and 15 studies were retained for the final analysis (**Figure 1**).

### 3.1 Study and model-related characteristics

Of the 15 studies, six were prospective (12–17), and seven were retrospective (18–24) cohort studies. Two studies (25, 26) considered data from the POCER study, a prospective randomised clinical trial, to investigate the optimal strategy for preventing recurrence after intestinal resection in patients with CD. Study sample sizes ranged between 18 and 1639 patients, with the percentage of female ranging from 21.9% to 76.9%. Stricturing disease behaviour was reported in 8% to 70.9% of patients.

Postoperative prophylactic treatments were described in all studies. Seven studies (13, 15, 17, 18, 20, 22, 24) believed that it was not a confounding factor based on the findings of univariate analysis, and did not include it in the final analysis. Four studies (12, 21, 25, 26) noted the limitations that an influence of postoperative medication on outcome could not be ruled out. In two studies (14, 23), patients were divided into two cohorts according to the postoperative biologic exposure to limit the confounding effect of biologic therapy. One study (16) incorporated treatment use between the second and third weeks postoperatively in the final model. The remaining one study (19) included immunomodulatory use in the final model. However, it failed to distinguish whether it preceded or followed the surgical recurrence. **Table 1** summarises the characteristics of each study, including study design, sample size, participant-related characteristics, and postoperative prophylactic treatments.

**Table 1:** Study characteristics.

Author (year)	Study design	Country	Sample size	Follow-up duration	Age	Female	Disease phenotype	Postoperative prophylactic treatments
Wu EH (2022) (18)	Retrospective single-center cohort	China	1639	≥12 months	<17 (11.5%) 17-40 (69.3%) >40 (19.2%)	31.4%	inflammatory (3.1%) stricturing (36.5%) penetrating (60.4%) perianal (26.9%)	Immunomodulators (81.1%) Corticosteroids (1.5%) Infliximab (15.8%) Other (1.6%)
Wang MH (2021) (19)	Retrospective	America	372	Not available	Cases: 26.5 ± 11.9 Controls: 29.4 ± 13.8	36.9%	Cases: inflammatory (17%) stricturing/penetrating (83%) Controls: inflammatory (17%) stricturing/penetrating (83%)	Immunomodulators Anti–TNF α No treatment (proportions unknown)
Primas C (2021) (12)	Prospective, single-center	Austria	67	Median: 349 days Range: 115-630 days	<17 (1.3%) 17-40 (79.7%) >40 (19%)	40.5%	inflammatory (29.1%) stricturing (70.9%) penetrating (34.2%)	Immunomodulators (83.6%) Anti–TNF α (11.4%) No treatment (5%)
Moret-Tatay I (2021) (13)	Prospective and consecutive cohort	Spain	32	Median: 12 months 1st-3rd quartile: 8.3-13.8 months	34.2 ± 13.0	21.9%	inflammatory (12.5%) stricturing (59.4%) penetrating (28.1%) perianal (21.9%)	Immunomodulators (59.4%) Anti–TNF α (21.9%) Combo therapy (3.1%) No treatment (15.6%)
Kusunoki K (2021) (20)	Retrospective cohort	Japan	100 for discovery; 169 for validation	Median: 72.5 months	≤ 37 (50%)	27%	inflammatory (41%) stricturing (8%) penetrating (51%)	Biologics (proportions unknown)
De Cruz P (2021) (25)	Part of the POCER study (a prospective randomised controlled trial)	Australia and New Zealand	85	18 months	<17 (9.4%) 17-40 (74.1%) >40 (16.5%)	49.4%	inflammatory (7.1%) stricturing (35.3%) penetrating (57.6%)	Immunomodulators Adalimumab (proportions unknown)
Akiyama S (2021) (21)	Retrospective, single-center	Chicago	127 (25 with colon-dominant; 102 with small intestine-dominant)	Median: 36 months Range: 0.6-109 months	38.1 ± 13.8	51.2%	inflammatory (9.2%) stricturing (39.1%) penetrating (14.5%) stricturing + penetrating (37.2%) perianal (28.5%)	Immunomodulators (70.0%) Corticosteroids (53.1%) Biologics (72.5%) Antibiotics (52.2%)
Sokol H (2020) (14)	Prospective multicentric cohort	France	201	7.9 ± 3.5 months	<17 (14%) 17-40 (75%) >40 (11%)	Not available	inflammatory (15%) stricturing (52%) penetrating (33%)	Immunomodulators (24.0%) Anti-TNF α(34.0%) Antibiotics (6.0%)
Machiels K (2020) (15)	Prospective, single-center cohort	Belgium	120	6 months	Remission: median 53.9 Recurrence: median 48.1	52.5%	Remission: stricturing (52.9%) penetrating (47.1%) perianal (13.2%) Recurrence: stricturing (57.7%) penetrating (42.3%) perianal (7.7%)	Immunomodulators (20.0%) Corticosteroids (20.8%) Anti-TNF α (18.3%) Antibiotics (17.5%)
Ikeda A (2019) (22)	Retrospective, single-center	Japan	52	6-12 months	Non-remission: median 41 (range 22–72) Remission: median 41 (range 24–68)	76.9%	Non-remission: penetrating/non-penetrating/perianal (16/21/14) Remission: penetrating/non-penetrating/perianal (10/5/3)	Immunomodulators (19.2%) Corticosteroids (5.7%) Anti-TNF α (55.8%) Elemental diet (100%)
Cushing KC (2019) (23)	Retrospective	America	60	Anti-TNFα-naïve: i0: 239 ± 10 days >i0: 258 ± 71 days	Anti-TNFα-naïve: 39 ± 14	45.8%	Anti-TNFα-naïve: inflammatory (8.3%) stricturing (41.7%) penetrating (50%)	Anti-TNF α (60.0%)
Cerrillo E (2019) (16)	Prospective, single-center	Spain	61	2 years	Mean 40.7 (range 18–74)	36.1%	inflammatory (29.5%) stricturing (41%) penetrating (29.5%) perianal (23%)	Immunomodulators (63.9%) Anti-TNFα (22.9%), Combo therapy (1.7%) No treatment (11.5%)
Auzoux J (2019) (17)	Prospective cohort	France	18	Median: 38 months Interquartile range: 20-41 months	38.1 ± 13.6	50%	inflammatory (5.6%) stricturing (38.9%) penetrating (55.6%) perianal (11.1%)	Immunomodulators (33.3%) Anti-TNF α (61.1%) Combo therapy (5.6%)
Nakao S (2017) (24)	Retrospective, single-center cohort	Japan	40	49.7 ± 34.7 months	>20, 34.2 ± 10.7	27.5%	penetrating (42.5%) non-penetrating (57.5%)	Immunomodulators (29.4%) Anti-TNF α (47.0%) Therapy intensification (43.1%)
Hamilton AL (2017) (26)	POCER study (Prospective, randomised, multi-center trial)	Australia	169	18 months	<17 (11%) 17-40 (76%) >40 (12%)	54%	inflammatory (9%) stricturing (36%) penetrating (55%)	Immunomodulators (59.0%) Antibiotics (16.0%) Adalimumab (25.0%)

CD, Crohn’s disease; TNF, tumour necrosis factor.

Twelve studies developed 15 new models for predicting CD postoperative recurrence (12–16, 18, 19, 22–26).. The other three studies validated the performance of existing models, namely the Watson score (17), advanced lung cancer inflammatory index (20), and simple endoscopic score for CD (SES-CD) (21), for predicting postoperative CD recurrence. Seven models used regression analysis (six using logistic regression (12, 13, 16, 19, 22, 24) and one Cox regression (18)), six used scoring systems (17, 20, 21, 25, 26), and five used machine learning algorithms (14, 15, 23) to predict CD recurrence. Predictors applied in prediction models were multifarious and included demographic or clinical characteristics, endoscopic or pathological manifestations, serological or faecal biomarkers, genetic factors, and gut microbiota. Six of the 15 studies reported internal validation findings (13–15, 18, 22, 25), while only one study reported external validation results (20). **Table 2** includes selected information describing prognostic models considered, including predictors, modelling methods, model performance, and validation. The main predictors of different types identified for endoscopic, surgical, and clinical recurrence were showed in **Supplementary Table 4**.

**Table 2:** Model information of included studies.

Author (year)	Outcome [Definition]	Candidate predictors	Modelling method	Model performance	Model evaluation
Wu EH (2022) (18)	Surgical recurrence [requiring repeat surgery for an indication related to CD]	23 clinical factors	Cox survival regression	Calibration curve C-index: 0.744 [95% CI 0.714–0.774]	Internal validation (bootstrap resampling)
Wang MH (2021) (19)	Surgical recurrence [having at least one resections after the first surgery secondary to CD complications]	14 clinical and genetic predictors	Logistic regression	AUC of ROC: Clinical + genetic: 0.87 Clinical only: 0.83 Cutoff: 3.59 Sensitivity 70%, Specificity 81%, PPV 67%, NPV 83%	None
Primas C (2021) (12)	Postoperative endoscopic recurrence at 12 months [Rutgeerts score ≥i2b]	Faecal calprotectin and 6 clinical factors	Logistic regression	AUC of ROC: 0.694 [95% CI 0.566–0.823] Cutoff: 2.54 Sensitivity 47.1%, Specificity 87.9%	None
Moret-Tatay I (2021) (13)	Postoperative morphological recurrence within 6-12 months [Rutgeerts score≥i2b or Sailer score≥MR2]	34 plasma miRNA	Elastic net penalised logistic regression	AUC of ROC: Development: 0.88 [95% CI 0.79-0.98] Internal validation: 0.88	Internal validation (bootstrap with 200 replicates)
Kusunoki K (2021) (20)	Surgical relapse within 5 years [requirement for CD-related surgery due to CD-related complications or refractoriness to treatments]	Not applicable	Cox proportional hazards analysis	AUC of ROC: 0.71 Cutoff: 19.93 Sensitivity 53%, Specificity 86%	External validation
De Cruz P (2021) (25)	At 18 months after surgery: Endoscopic recurrence [Rutgeert score≥i2]; Mucosal healing [Rutgeerts score = i0]	5 endoscopic parameters	Stepwise addition of variables together with bootstrapping	Endoscopic recurrence: AUC of ROC: 0.70 [95% CI 0.57-0.82] Cutoff: 2 Sensitivity: 80.4% [95% CI, 67.6%-89.8%] Specificity: 41.4% [95% CI, 23.5%-61.8%] PPV: 72.6% [95% CI, 59.8%-83.1%] NPV: 52.2% [95% CI, 30.6%-73.2%] Complete mucosal healing: Sensitivity: 57.7% [95% CI, 36.9%-76.6%] Specificity: 72.9% [95% CI, 59.7%-83.6%] PPV: 48.4% [95% CI, 30.2%-66.9%] NPV: 79.6% [95% CI, 66.5%-89.4%]	Monte Carlo Markov simulation with 100 repetitions
Akiyama S (2021) (21)	Postoperative clinical recurrence [Harvey–Bradshaw index>4]	Not applicable	Not applicable	AUC of ROC: 0.70 [95% CI 0.60–0.79] Cutoff: 2 Sensitivity 71.1%, Specificity 58.5% Small intestine-dominant disease: AUC of ROC: 0.67 [95% CI 0.56–0.78] Cutoff: 1 Colon-dominant disease: AUC of ROC: 0.76 [95% CI 0.57–0.95] Cutoff: 5	None
Sokol H (2020) (14)	Postoperative endoscopic recurrence about 6-12 months [Rutgeerts score ≥ i2]	9 gut microbiota taxa and 3 clinical factors	Random forest	AUC of ROC: Microbiota factors alone: whole population: 0.971 [95% CI 0.938–1] validation set: 0.81 [95% CI 0.608–1] Microbiota+clinical factors: whole population: 0.98 [95% CI 95.6–1] validation set: 0.786 [95% CI 0.569–1]	Internal validation (random split)
Machiels K (2020) (15)	Postoperative endoscopic recurrence at 6 months [Rutgeerts score ≥i2b]	Faecal/mucosal microbial factors, clinical factors	Decision tree (C5.0 algorithm)	AUC of ROC: Mucosal: C5.0: clinical 0.612, microbiota 0.738, combination 0.779 Random forest: clinical 0.651, microbiota 1, combination 1 Faecal: C5.0: clinical 0.5, microbiota 0.79, combination 0.79 Random forest: clinical 0.375, microbiota 0.5, combination 0.5	Internal validation (random forest)
Ikeda A (2019) (22)	Postoperative endoscopic recurrence within 6-12 months [Rutgeert score≥i2b]	Clinical factors, perioperative medications, laboratory findings	Logistic regression	AUC of ROC: 0.808	Internal validation
Cushing KC (2019) (23)	Anti-TNF-Naïve Cohort: differential classification of Rutgeerts score i0 vs i1-i4 of the first postoperative endoscopy [i0: complete mucosal remission]; Anti-TNF-Exposed Cohort: differential classification of aggressive [a composite score≥14] and indolent [score ≤ 8] disease	Transcripts of mucosal biopsies	Random forest, classification	an out-of-bag estimate of error rate: Anti-TNF-Naïve: 8.33% Anti-TNF-Exposed: 7.14%	None
Cerrillo E (2019) (16)	Postoperative morphological recurrence within 6-12 months: Endoscopic recurrence [Rutgeert score≥i2b] Radiological recurrence [Sailer index≥MR2]	16 faecal, clinical, demographic, serological variables	Logistic regression	AUC of ROC: 0.90 [95% CI 0.76-1]	None
Auzoux J (2019) (17)	Within 6-12 months: Postoperative endoscopic recurrence [Rutgeerts≥i2] Postoperative clinical relapse [an elevated faecal calprotectin level (>250 mg/g stool), and the increase or occurrence of symptoms associated with a Harvey-Bradshaw index score >5 at 2 examinations 7 days apart]	Not applicable	Not applicable	Endoscopic recurrence: AUC of ROC: 0.766 (95% CI 0.550-0.983) Cutoff: 2 Sensitivity 71%, Specificity 82%, PPV 71%, NPV 82%, Accuracy 78% Symptomatic recurrence: Cutoff: 2 Sensitivity 80%, Specificity 77%, PPV 91%, NPV 57%, Accuracy 78%	None
Nakao S (2017) (24)	Postoperative endoscopic recurrence [Rutgeerts score≥i2 confined to the anastomotic site]	49 clinical and pathological variables	Logistic regression	R-squared: 0.598 AUC of ROC: 0.934	None
Hamilton AL (2017) (26)	Postoperative endoscopic recurrence at 6 or 18 months [Rutgeert score≥i2]	8 serological antibodies	Calculation for quartile sum score and number of positive markers	AUC of ROC: Total quartile sum score: 0.50 for 6 month 0.60 for 18 month Total number of positive antibodies: 0.51 for 6 month 0.56 for 18 month	None

AUC, area under the curve; CD, Crohn’s disease; CI, confidence interval; NPV, negative predictive value; PPV, positive predictive value; ROC, receiver operating characteristic curve; TNF, tumour necrosis factor.

### 3.2 Risk of bias

**Figure 2:**
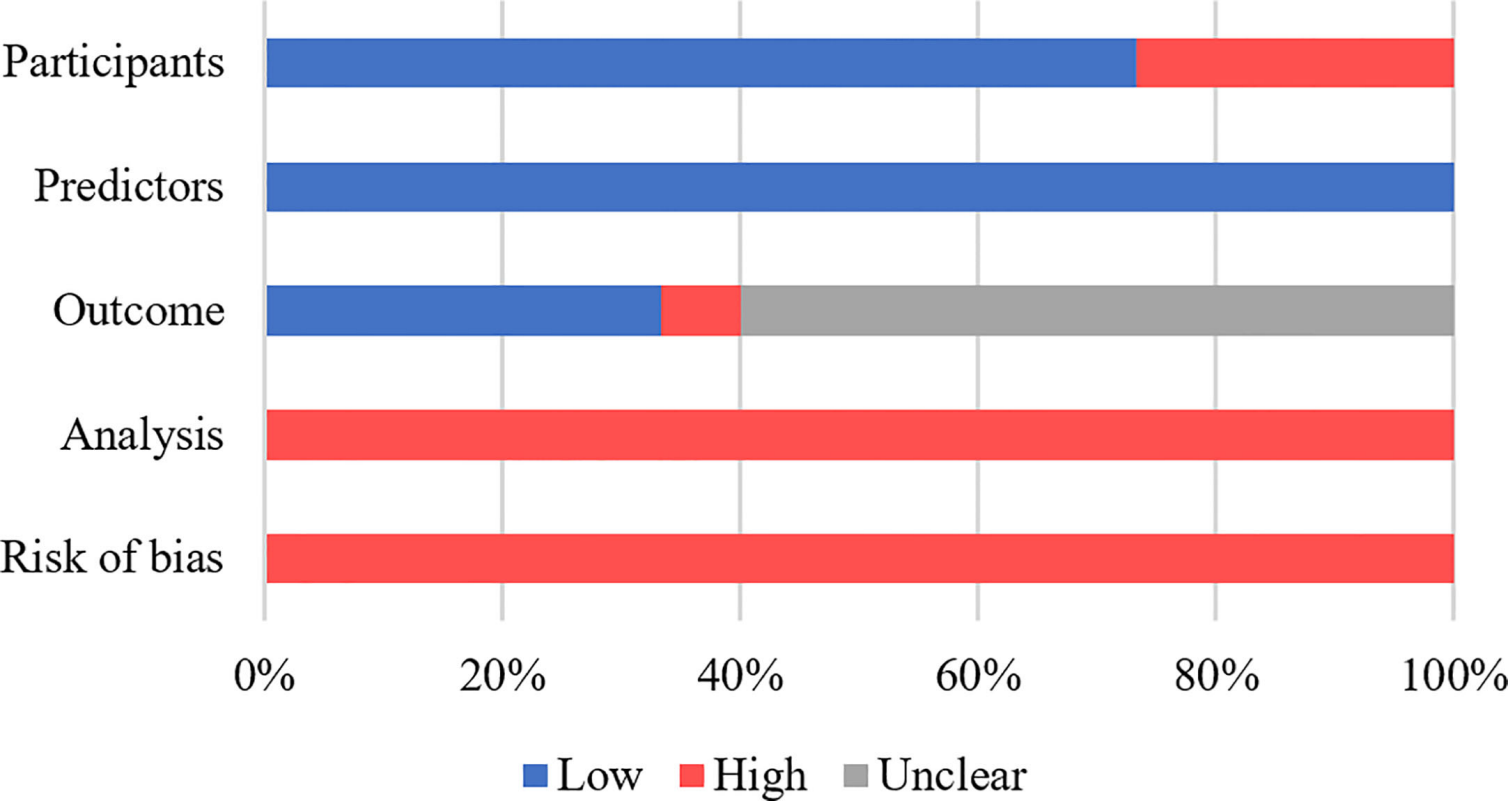
Risk of bias assessment.

In **Supplementary Table 3**, the evaluation of the ROB and applicability of each study throughout four domains (participants, predictors, outcomes, and analysis) are shown. All studies had a high ROB (**Figure 2**), with the greatest degree of risk arising from the analysis domain, which was mainly attributed to small sample sizes, insufficient information on handling missing data, failure to assess model calibration, a lack of internal validation, or incomplete reporting of the internal validation process. For participants domain, only four studies were considered at high ROB, as the studies excluded participants with incomplete data or lack of follow-up, potentially introducing selection bias (13, 18, 20, 22). Information regarding predictors of CD recurrence was reported relatively clearly across all studies, and therefore, resulted in a low ROB. Regarding the outcome domain, ten studies failed to indicate whether the outcome was evaluated without knowledge of predictor information (14, 15, 17–19, 22–26), while one study (19) failed to clarify the specific timeframe used for outcome determination. In the study by Cushing et al. (23), the outcome domain was determined to be associated with a high ROB because the adopted outcome threshold was derived using an unsupervised hierarchical clustering algorithm rather than a generally accepted classification system. All the studies were at low risk of applicability concerns (**Supplementary Table 3**), because the explicit purpose of this systematic review was to assess models with potential prognostic value for postoperative CD recurrence, with less emphasis placed on the heterogeneity of participants, predictors, outcomes, and analysis methods.

### 3.3 Prognostic models for postoperative recurrence

Based on the definition of outcomes, we divided prognostic models for predicting postoperative recurrence of CD into the following three broad categories: postoperative endoscopic recurrence, postoperative surgical recurrence, and postoperative clinical recurrence.

#### 3.3.1 Predicting endoscopic recurrence

**Figure 3:**
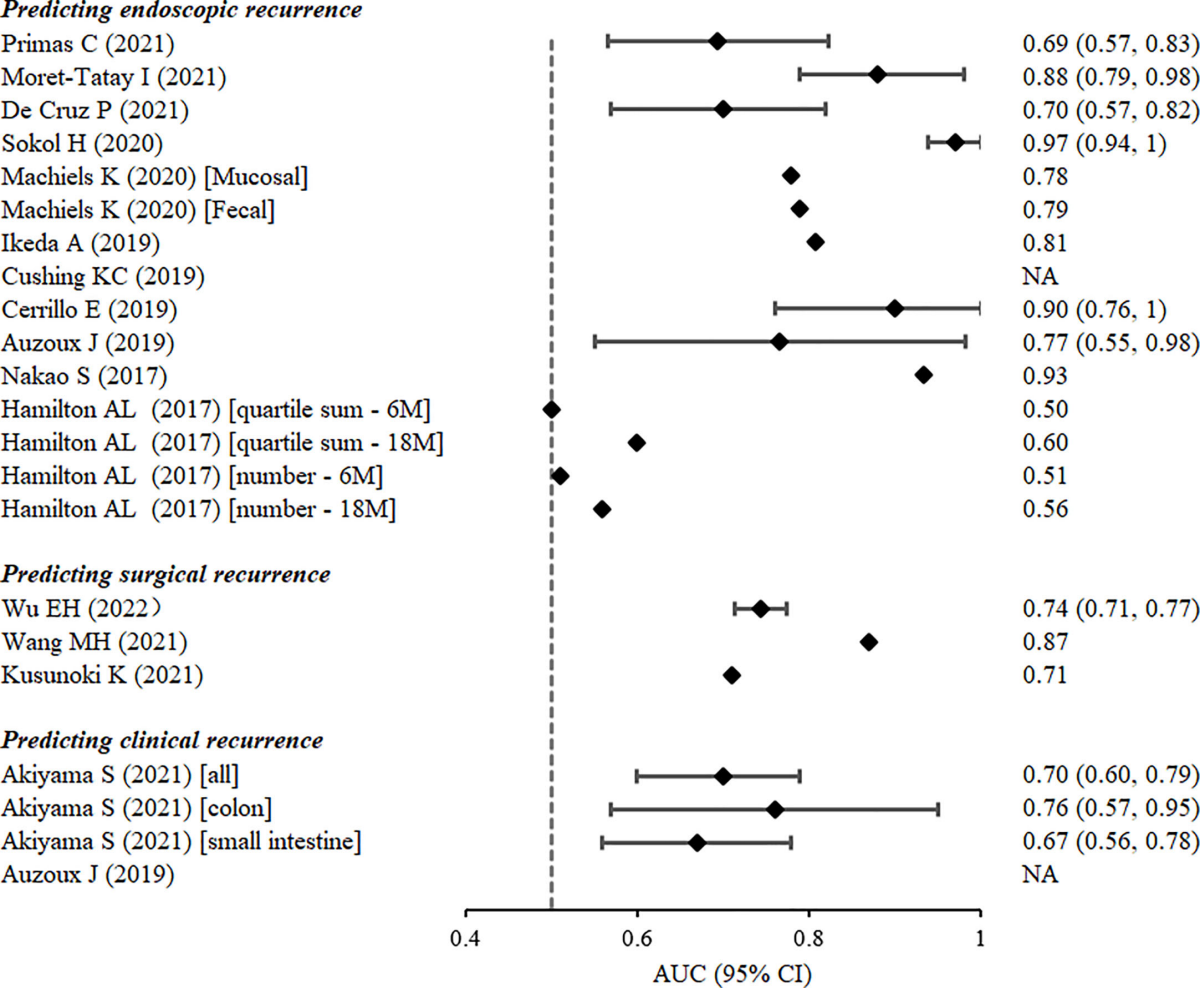
Discrimination of prognostic models estimated by the area under the receiver-operating characteristic curve from the derivation cohorts.

Eleven studies addressed 11 prognostic models for CD-related postoperative endoscopic recurrence. AUC values of the prognostic models ranged from 0.51 to 0.97 (**Figure 3**). Predictors included endoscopic parameters, pathological parameters, clinical factors, serological factors, faecal calprotectin levels, and gut microbiota.

Cushing et al. (23) divided patients into two cohorts according to their exposure to anti-tumour necrosis factor (TNF). A random forest model of transcripts within mucosal biopsies was constructed in anti-TNF-naïve cohort to distinguish postoperative endoscopic recurrence (Rutgeerts’ score ≥i1) from mucosal healing. An out-of-bag estimate of the error rate, rather than the AUC value, was reported as 8.33%. However, in the anti-TNF-exposed cohort, CD disease activity was classified as indolent or aggressive based on a self-constructed composite score. The out-of-bag estimate of the error rate for the classification of postoperative disease activity was 7.14%.

The 10 remaining studies defined postoperative endoscopic recurrence as a Rutgeerts’ score ≥i2 or modified Rutgeerts’ score ≥i2b. One study reported a Watson score, which was composed of confocal laser endoscopic parameters at 6–12 months after ileocolectomy, as a useful tool for predicting the subsequent endoscopic recurrence of CD (AUC: 0.766, 95% CI: 0.550–0.983) (17). Five studies used logistic regression analysis to construct prognostic models (12, 13, 16, 22, 24), four of which presented AUC values >0.80. Nakao et al. (24) confirmed the prognostic value of myenteric and submucosal plexitis for predicting the postoperative recurrence of CD, and developed a logistic regression model composed of both clinical and pathological variables with an AUC value as high as 0.934. Three nomograms were constructed to predict endoscopic recurrence 6–12 months after surgery in patients with CD. One of the nomograms of five plasma miRNAs predicted postoperative CD recurrence with a high degree of accuracy (AUC: 0.88, 95% CI: 0.79–0.98) (13). The nomogram with three predictors, namely excessive perioperative inflammation, number of previous CD-related intestinal resections, and levels of preoperative serum albumin, also had a satisfactory potential for predicting endoscopic recurrence (AUC: 0.808) (22). Another nomogram that assessed risk of endoscopic recurrence by considering combined levels of faecal calprotectin, interleukin-6, and interferon-γ, as well as the presence of postoperative prophylactic therapy 6 months postoperatively, exhibited remarkable apparent performance (AUC: 0.90, 95% CI: 0.76–1) (16). Of the four remaining studies, two reported machine-learning models based on the gut microbiota. Machiels et al. (15) developed a C5.0 classification decision tree based on the mucosal microbiota of patients with CD at resection to predict endoscopic recurrence 6 months after surgery in a prospective study. The decision tree composed of mucosal *Phasolarctobacterium*, *Gemella*, *Haemophilus*, and *Ralstonia* abundance had better discriminative power for predicting postoperative recurrence (AUC: 0.738) than that of the model that considered clinical factors (AUC: 0.612). Sokol et al. (14) created a random forest model based on nine gut microbiotas to predict endoscopic recurrence 6–12 months after CD-related surgery. The model exhibited excellent predictive ability in the validation set (AUC: 0.81, 95% CI: 0.608–1); however, adding clinical predictors did not improve model performance. The other two studies constructed simple prognostic scores by the stepwise addition of variables and the calculation of the sums of quartiles and the number of positive markers, respectively. Nevertheless, both scores have limited discriminatory power (25, 26).

#### 3.3.2 Predicting surgical recurrence

Three prognostic models were reported for predicting surgical recurrence, the need for reoperation after surgery due to CD-related indications or complications. The discriminatory power of the models was assessed by the AUC, which ranged from 0.71 to 0.87 (**Figure 3**). Predictors considered included clinical and genetic factors. Wu et al. (18) constructed a nomogram with satisfactory calibration and accuracy (AUC 0.744; 95% CI 0.714–0.774). Four variables were included, namely staged surgery, penetration behaviour, upper gastrointestinal disease, and emergency at initial surgery. In addition, Kusunoki et al. (20) found that the advanced lung cancer inflammation index, a proposed nutritional index composed of body mass index, preoperative serum albumin levels, and neutrophil-to-lymphocyte ratio, was an independent predictor with modest accuracy (AUC 0.71) of postoperative surgical relapse within five years in patients with CD. Another study (19), however, considered both clinical and genetic risk factors. By logistic forward stepwise regression, the researchers developed a composite score with the following three variables: an early era of the first CD-related surgery, history of immunomodulatory agent use, and genetic locus rs2060886 in transcription factor-4. This risk score demonstrated superior predictive power over clinical factors alone for predicting surgical recurrence, with an AUC of 0.87.

#### 3.3.3 Predicting clinical recurrence

Two studies independently validated the predictive power of two existing endoscopic variable-based scores for clinical recurrence after CD-related surgery. Akiyama et al. (21) evaluated the performance of SES-CD, an index that utilises endoscopic CD activity at the first postoperative ileocolonoscopy to predict postoperative clinical recurrence, which was defined as a Harvey–Bradshaw index score >4.The median interval from surgery to clinical recurrence was 36 months. It was demonstrated that SES-CD had acceptable discriminatory power regarding the prediction of clinical recurrence in colon-dominant CD (AUC: 0.76, 95% CI: 0.57–0.95); however, its ability to predict small intestine-dominant disease was relatively poor (AUC: 0.67, 95% CI: 0.56–0.78). Auzoux et al. (17) assessed the prognostic potential of the Watson score, with a median follow-up of 38 months. Study findings showed that a Watson score with a cutoff value of 2 was predictive of postoperative symptomatic recurrence, with a sensitivity and specificity of 80% and 77%, respectively.

## 4 Discussion

In this systematic review, we characterised 15 published prognostic models for predicting postoperative CD recurrence and assessed their ROB and applicability. We found that traditional regression models accounted for a large proportion of existing models. A rise in the popularity of machine learning-based algorithms and various omics technologies has also generated new ideas for developing multivariate models. Predictors considered in these models included clinical characteristics, medication history, laboratory test results, endoscopic variables, pathological parameters, gut microbiota, and genetic factors. This was consistent with independent risk factors of postoperative CD recurrence proposed in previous studies (3, 27–30). Current guidelines recommend prophylactic treatment for patients with CD who are at high risk of postoperative recurrence according to their clinical characteristics (31, 32). However, such risk stratification is insufficient because a single, reliable prognostic indicator of CD recurrence has yet to be identified. Therefore, the prognostic models highlighted in this systematic review are of clinical value and have the potential to inform future clinical research.

Although it appears that the prognostic value of models included in our systematic review requires further verification before they may be used in a clinical setting, model development studies provided many insights. First, many studies have shown that models incorporating different types of predictors outperform those that include clinical factors alone, suggesting that predictor diversity should be considered in future models. Second, in addition to traditional regression models, machine learning techniques, although limited in interpretability and clinical utility due to their complexity, have shown great potential for identifying informative predictors for succeeding modelling. Third, large prospective studies are preferred for model development. A small sample size accompanied by numerous candidate variables for modelling may result in the overestimation of apparent model performance (33–35). However, a prospective design allows for better control of study conditions. For example, a fixed time for outcome determination may be ensured, thereby reducing heterogeneity and increasing model performance reliability. Fourth, to validate model performance and applicability, published prognostic models should be externally validated using large, representative cohorts, with models updated, if necessary. The evaluation of the clinical utility and feasibility of implementation of models is also required before they can be used in clinical practice. Finally, problems such as vague reports and the lack of standardised and rigorous modelling procedures in existing studies are best avoided. We recommend that prognostic models are created and reported strictly in accordance with the Transparent Reporting of a multivariable prediction model for Individual Prognosis Or Diagnosis (TRIPOD) guidelines (36) to prevent bias and spurious predictor-outcome associations, and facilitate subsequent interpretation and validation of the models.

A limitation of this systematic review is that subjective assessments may be introduced during ROB and applicability evaluation, even though PROBAST was used. This is mainly because PROBAST allows reviewers to make subjective judgments outside the signal questions. In addition, studies identified in this review were limited by their high ROB due to methodological flaws. Further, a great degree of heterogeneity across studies existed in terms of research conditions, variables, and predicted outcomes. Specifically, the definitions of endoscopic, surgical, and clinical recurrence varied widely across studies. On the one hand, the interval from surgery to the recurrence evaluation ranged from within 6 months to 5 years; on the other hand, different studies applied different scores or indicators as standards. Thus, we could not conclusively state which model was superior to the others. But we believe that the emergence of these models did represent a trend in which the ability of clinical factors to predict postoperative recurrence was unsatisfactory, and exploring some new easily accessible microbiological, pathological, and genetic variables is more promising for the construction of impactful predictive models. In particular, using machine learning techniques such as decision tree and random forest to screen novel predictors based on omics may be more valuable of attention and attempts, provided that prospective studies in large cohorts and effective external validation are conducted.

In general, constructing a practical prediction model for postoperative risk stratification is of great importance, which will help clinicians formulate and adjust follow-up treatment strategies for patients with CD after surgery in a timely manner. More prognostic models of clinical significance are needed, and increased attention and investment is recommended.

## Data availability statement

The original contributions presented in the study are included in the article/**Supplementary Material**. Further inquiries can be directed to the corresponding authors.

## Author contributions

Guarantor of article: SZ. SZ and MC: Conceptualization; funding acquisition; writing- review & editing. RC: Formal analysis; methodology; writing-original draft; project administration. JZ: Data curation; formal analysis; writing-original draft; project administration. CL and QC: Data curation; project administration. LL and ZZ: supervision; validation. All authors contributed to the article and approved the submitted version.
