# Supplementary material for: Prognostic models for predicting postoperative recurrence in Crohn’s disease: a systematic review and critical appraisal
Source: Front Immunol. 2023 Jun 30;14:1215116. doi: 10.3389/fimmu.2023.1215116 (PMC10349525; doi:10.3389/fimmu.2023.1215116)
Supplement: Supplementary file 1 [file DataSheet_1.docx]

Supplementary Material

Prognostic models for predicting postoperative recurrence in Crohn's disease: a systematic review and critical appraisal

Rirong Chen^†^, Jieqi Zheng^†^, Chao Li, Qia Chen, Zhirong Zeng, Li Li, Minhu Chen, Shenghong Zhang

†These authors contributed equally to this work and share first authorship

*** Correspondence:**Shenghong Zhang: [shenghongzhang@163.com](mailto:shenghongzhang@163.com); [zhshh3@mail.sysu.edu.cn](mailto:zhshh3@mail.sysu.edu.cn)
Minhu Chen: [chenminhu@mail.sysu.edu.cn](mailto:chenminhu@mail.sysu.edu.cn)

# Supplementary Table 1 Search strategy

| **PubMed** | |
| --- | --- |
|  | Search: ((((((((((((((((((((((Validat*) OR (Predict*[Title])) OR (Rule*)) OR ((Predict* AND (Outcome* OR Risk* OR Model*)))) OR (((History OR Variable* OR Criteria OR Scor* OR Characteristic* OR Finding* OR Factor*) AND (Predict* OR Model* OR Decision* OR Identif* OR Prognos*)))) OR ((Decision* AND (Model* OR Clinical* OR Logistic Models/)))) OR (Prognostic AND (History OR Variable* OR Criteria OR Scor* OR Characteristic* OR Finding* OR Factor* OR Model*))) OR ("Stratification")) OR ("ROC Curve"[MeSH Terms])) OR ("Discrimination")) OR ("Discriminate")) OR ("c-statistic")) OR ("c statistic")) OR ("Area under the curve")) OR ("AUC")) OR ("Calibration")) OR ("Indices")) OR ("Algorithm")) OR ("Multivariable")) AND (((((((((postoperative) OR (postsurgical)) OR (resection)) OR (colectomy)) OR (surgery)) OR (operation)) OR (ileocolectomy)) OR (ileocectomy)) OR (ileectomy))) AND (relapse OR recurrence)) AND (((Crohns Disease[Title/Abstract]) OR (Crohn disease[Title/Abstract])) OR (Crohn’s disease[Title/Abstract]))) NOT ((((((((((((((((((((((Validat*) OR (Predict*[Title])) OR (Rule*)) OR ((Predict* AND (Outcome* OR Risk* OR Model*)))) OR (((History OR Variable* OR Criteria OR Scor* OR Characteristic* OR Finding* OR Factor*) AND (Predict* OR Model* OR Decision* OR Identif* OR Prognos*)))) OR ((Decision* AND (Model* OR Clinical* OR Logistic Models/)))) OR (Prognostic AND (History OR Variable* OR Criteria OR Scor* OR Characteristic* OR Finding* OR Factor* OR Model*))) OR ("Stratification")) OR ("ROC Curve"[MeSH Terms])) OR ("Discrimination")) OR ("Discriminate")) OR ("c-statistic")) OR ("c statistic")) OR ("Area under the curve")) OR ("AUC")) OR ("Calibration")) OR ("Indices")) OR ("Algorithm")) OR ("Multivariable")) AND (((((((((postoperative) OR (postsurgical)) OR (resection)) OR (colectomy)) OR (surgery)) OR (operation)) OR (ileocolectomy)) OR (ileocectomy)) OR (ileectomy))) AND (relapse OR recurrence)) AND (((Crohns Disease[Title/Abstract]) OR (Crohn disease[Title/Abstract])) OR (Crohn’s disease[Title/Abstract])) AND (guideline[Filter] OR meta-analysis[Filter] OR review[Filter] OR systematicreview[Filter])) |
|  | Results: N=1013 |
| **Web of Science** | |
| #1 | (((((((((((((((ALL=(Validat*)) OR TI=(Predict*)) OR ALL=(Rule*)) OR ALL=((Predict* AND (Outcome* OR Risk* OR Model*)))) OR ALL=(((History OR Variable* OR Criteria OR Scor* OR Characteristic* OR Finding* OR Factor*) AND (Predict* OR Model* OR Decision* OR Identif* OR Prognos*)))) OR ALL=(Decision* AND (Model* OR Clinical* OR Logistic ))) OR ALL=(Prognostic AND (History OR Variable* OR Criteria OR Scor* OR Characteristic* OR Finding* OR Factor* OR Model*))) OR ALL=("Discriminate")) OR ALL=("c-statistic")) OR ALL=("c statistic")) OR ALL=("Area under the curve")) OR ALL=("AUC")) OR ALL=("Calibration")) OR ALL=("Indices")) OR ALL=("Algorithm")) OR ALL=("Multivariable") |
| #2 | ((((((((ALL=(postoperative)) OR ALL=(postsurgical)) OR ALL=(resection)) OR ALL=(colectomy)) OR ALL=(surgery)) OR ALL=(operation)) OR ALL=(ileocolectomy)) OR ALL=(ileocectomy)) OR ALL=(ileectomy) |
| #3 | (ALL=(relapse)) OR ALL=(recurrence) |
| #4 | ((TS=(Crohn’s disease)) OR TS=(Crohn disease)) OR TS=(Crohns Disease) |
| #5 | #1 AND #2 AND #3 AND #4 and Review Articles or Meeting Abstracts or Reprints or Editorial Materials (Exclude – Document Types) |
|  | Results: N=935 |

# Supplementary Table 2 Assessment of risk of bias and applicability based on PROBAST

| 1. **Wu EH et al, 2022. Patients with Crohn's Disease Undergoing Abdominal Surgery: Clinical and Prognostic Evaluation Based on a Single-Center Cohort in China.** | | | | | |
| --- | --- | --- | --- | --- | --- |
| **Domain 1: Participants** | | | | | |
| *The data sources and the criteria for participant selection:*  observational, retrospective single-center cohort analysis, medical record-based study.  The patients who underwent at least one abdominal surgery due to CD at the Center for inflammatory bowel diseases in the department of General surgery, Jinling Hospital of Nanjing University, from January 1, 2007, to December 31, 2020, were screened. Inclusion criteria: (1) The diagnosis of CD was based on clinical, ileocolonoscopic, histopathologic, and radiologic findings. Postoperative pathological results should be consistent with CD. (2) The first operation was performed in our center.  Exclusion criteria: (1) Patients with perianal surgery or abscess drainage. (2) Non-calculation was performed due to postoperative complications such as postoperative bleeding, anastomotic leakage, abdominal abscess, and complications of ostomy. (3) Lack of follow-up or in complete clinical data. | | | | | |
| **I. Risk of Bias** | | | | | |
| Signaling questions | 1. Were appropriate data sources used, e.g., cohort, randomized controlled trial, or nested case-control study data? | | | | Y |
|  | 2. Were all inclusions and exclusions of participants appropriate? | | | | PN |
| Bias rating | *Rationale of bias rating:*  Patients lack of follow-up or in complete clinical data were excluded. | | | | **high** |
| **II. Applicability** | | | | | |
| Concern that included participants or the setting do not match the review question | | Applicability rating | | | **low** |
|  |  | *Rationale of applicability rating:* | | | |
| **Domain 2: Predictors** | | | | | |
| *The support for judgment box：*  Clinical factors; four variables [upper gastrointestinal disease, penetrating behavior, emergency at initial surgery, staged surgery at the initial abdominal surgery] were included into final model. | | | | | |
| **I. Risk of Bias** | | | | | |
| Signaling questions | 1. Were predictors defined and assessed in a similar way for all participants? | | | | Y |
|  | 2. Were predictor assessments made without knowledge of outcome data? | | | | Y |
|  | 3. Are all predictors available at the time the model is intended to be used? | | | | Y |
| Bias rating | *Rationale of bias rating:* | | | | **low** |
| **II. Applicability** | | | | | |
| Concern that included participants or the setting do not match the review question | | Applicability rating | | | **low** |
|  |  | *Rationale of applicability rating:* | | | |
| **Domain 3: Outcome** | | | | | |
| *The support for judgment box：*  Outcome: surgical recurrence [the requirement for repeat surgery for a CD-related indication] | | | | | |
| **I. Risk of Bias** | | | | | |
| Signaling questions | 1. Was the outcome determined appropriately? | | | | Y |
|  | 2. Was a prespecified or standard outcome definition used? | | | | Y |
|  | 3. Were predictors excluded from the outcome definition? | | | | Y |
|  | 4. Was the outcome defined and determined in a similar way for all participants? | | | | Y |
|  | 5. Was the outcome determined without knowledge of predictor information? | | | | NI |
|  | 6. Was the time interval between predictor assessment and outcome determination appropriate? | | | | PY |
| Bias rating | *Rationale of bias rating:* | | | | **unclear** |
| **II. Applicability** | | | | | |
| Concern that included participants or the setting do not match the review question | | Applicability rating | | | **low** |
|  |  | *Rationale of applicability rating:* | | | |
| **Domain 4: Analysis** | | | | | |
| *The support for judgment box：*  Events per variable: 24  Modelling method: Nomogram plotted by using Cox survival regression models  Selection of predictors during modelling: The nomogram was established by using the step-down method, where all variables were included at first, and nonpredictive variables were removed to improve the accuracy of the predicted model.  Model performance: calibration curve, C-index  Model evaluation: internal validation (bootstrap resampling) | | | | | |
| **Risk of Bias** | | | | | |
|  | | | | | Dev |
| Signaling questions | 1. Were there a reasonable number of participants with the outcome? | | | | PY |
|  | 2. Were continuous and categorical predictors handled appropriately? | | | | Y |
|  | 3. Were all enrolled participants included in the analysis? | | | | Y |
|  | 4. Were participants with missing data handled appropriately? | | | | Y |
|  | 5. Was selection of predictors based on univariable analysis avoided? | | | | Y |
|  | 6. Were complexities in the data (e.g., censoring, competing risks, sampling of control participants) accounted for appropriately? | | | | PN |
|  | 7. Were relevant model performance measures evaluated appropriately? | | | | Y |
|  | 8. Were model overfitting and optimism in model performance accounted for? | | | | PY |
|  | 9. Do predictors and their assigned weights in the final model correspond to the results from the reported multivariable analysis? | | | | Y |
| Bias rating | *Rationale of bias rating:*  A short follow-up time (<12 months) among the patients whose first surgeries were conducted after December 2019 was inevitable. | | | | **high** |
| **Overall Assessment of Risk of Bias and Concerns for Applicability** | | | | | |
| Risk of bias | | | **high** | *Summary:*  Patients lack of follow-up or in complete clinical data were excluded, and the follow-up time was inconsistent. | |
| Applicability concerns | | | **low** | *Summary:* | |
|  | | | | | |
| 1. **Wang MH et al, 2021. Novel Genetic Variant Predicts Surgical Recurrence Risk in Crohn's Disease Patients.** | | | | | |
| **Domain 1: Participants** | | | | | |
| *The data sources and the criteria for participant selection:*  retrospective, two independent genetic association study data sets.  Adult patients (18 years and older) diagnosed with IBD and validated by medical record review were eligible. | | | | | |
| **I. Risk of Bias** | | | | | |
| Signaling questions | 1. Were appropriate data sources used, e.g., cohort, randomized controlled trial, or nested case-control study data? | | | | Y |
|  | 2. Were all inclusions and exclusions of participants appropriate? | | | | Y |
| Bias rating | *Rationale of bias rating:* | | | | **low** |
| **II. Applicability** | | | | | |
| Concern that included participants or the setting do not match the review question | | Applicability rating | | | **low** |
|  |  | *Rationale of applicability rating:* | | | |
| **Domain 2: Predictors** | | | | | |
| *The support for judgment box：*  clinical and genetic predictors | | | | | |
| **I. Risk of Bias** | | | | | |
| Signaling questions | 1. Were predictors defined and assessed in a similar way for all participants? | | | | Y |
|  | 2. Were predictor assessments made without knowledge of outcome data? | | | | NI |
|  | 3. Are all predictors available at the time the model is intended to be used? | | | | Y |
| Bias rating | *Rationale of bias rating:*  Use of objective predictors not requiring subjective interpretation. | | | | **low** |
| **II. Applicability** | | | | | |
| Concern that included participants or the setting do not match the review question | | Applicability rating | | | **low** |
|  |  | *Rationale of applicability rating:* | | | |
| **Domain 3: Outcome** | | | | | |
| *The support for judgment box：*  Outcome: surgical recurrence [having 1 or more resections after the first abdominal surgery secondary to complications of CD at the time of study enrollment] | | | | | |
| **I. Risk of Bias** | | | | | |
| Signaling questions | 1. Was the outcome determined appropriately? | | | | Y |
|  | 2. Was a prespecified or standard outcome definition used? | | | | Y |
|  | 3. Were predictors excluded from the outcome definition? | | | | Y |
|  | 4. Was the outcome defined and determined in a similar way for all participants? | | | | Y |
|  | 5. Was the outcome determined without knowledge of predictor information? | | | | NI |
|  | 6. Was the time interval between predictor assessment and outcome determination appropriate? | | | | NI |
| Bias rating | *Rationale of bias rating:* | | | | **unclear** |
| **II. Applicability** | | | | | |
| Concern that included participants or the setting do not match the review question | | Applicability rating | | | **low** |
|  |  | *Rationale of applicability rating:* | | | |
| **Domain 4: Analysis** | | | | | |
| *The support for judgment box：*  Events per variable: 9  Modelling method: multivariate logistic regression  Selection of predictors for inclusion: Genetic factors were selected based on univariate analysis.  Selection of predictors during modelling: a multivariable forward stepwise selection process  Model performance: the area under the curve of receiver operator characteristic curve [AUC of ROC], sensitivity, specificity, positive predictive value, negative predictive value | | | | | |
| **Risk of Bias** | | | | | |
| Signaling questions | 1. Were there a reasonable number of participants with the outcome? | | | | N |
|  | 2. Were continuous and categorical predictors handled appropriately? | | | | Y |
|  | 3. Were all enrolled participants included in the analysis? | | | | Y |
|  | 4. Were participants with missing data handled appropriately? | | | | Y |
|  | 5. Was selection of predictors based on univariable analysis avoided? | | | | N |
|  | 6. Were complexities in the data (e.g., censoring, competing risks, sampling of control participants) accounted for appropriately? | | | | PN |
|  | 7. Were relevant model performance measures evaluated appropriately? | | | | PY |
|  | 8. Were model overfitting and optimism in model performance accounted for? | | | | NI |
|  | 9. Do predictors and their assigned weights in the final model correspond to the results from the reported multivariable analysis? | | | | Y |
| Bias rating | *Rationale of bias rating:*  Events per variable is <10. Genetic factors were selected based on univariate analysis prior to multivariable modeling. No internal validation. No treatment information. | | | | **high** |
| **Overall Assessment of Risk of Bias and Concerns for Applicability** | | | | | |
| Risk of bias | | | **high** | *Summary:*  Unclear definition of the time interval between predictor assessment and outcome determination, small sample size, selection of predictors based on univariable analysis, no internal validation, no treatment information. | |
| Applicability concerns | | | **low** | *Summary:* | |
|  | | | | | |
| 1. **Primas C et al, 2021.** **Role of fecal calprotectin in predicting endoscopic recurrence in postoperative Crohn's disease.** | | | | | |
| **Domain 1: Participants** | | | | | |
| *The data sources and the criteria for participant selection:*  prospective, single-center study.  Patients with an established diagnosis of CD according to ECCO guidelines who had undergone a CD-related intestinal resection with ileocolonic anastomosis were prospectively enrolled. | | | | | |
| **I. Risk of Bias** | | | | | |
| Signaling questions | 1. Were appropriate data sources used, e.g., cohort, randomized controlled trial, or nested case-control study data? | | | | Y |
|  | 2. Were all inclusions and exclusions of participants appropriate? | | | | Y |
| Bias rating | *Rationale of bias rating:* | | | | **low** |
| **II. Applicability** | | | | | |
| Concern that included participants or the setting do not match the review question | | Applicability rating | | | **low** |
|  |  | *Rationale of applicability rating:* | | | |
| **Domain 2: Predictors** | | | | | |
| *The support for judgment box：*  Fecal calprotectin measured 6 months, clinical factors | | | | | |
| **I. Risk of Bias** | | | | | |
| Signaling questions | 1. Were predictors defined and assessed in a similar way for all participants? | | | | Y |
|  | 2. Were predictor assessments made without knowledge of outcome data? | | | | Y |
|  | 3. Are all predictors available at the time the model is intended to be used? | | | | Y |
| Bias rating | *Rationale of bias rating:* | | | | **low** |
| **II. Applicability** | | | | | |
| Concern that included participants or the setting do not match the review question | | Applicability rating | | | **low** |
|  |  | *Rationale of applicability rating:* | | | |
| **Domain 3: Outcome** | | | | | |
| *The support for judgment box：*  Outcome: 12-month postoperative endoscopic recurrence (Rutgeerts score ≥i2b), blind assessment | | | | | |
| **I. Risk of Bias** | | | | | |
| Signaling questions | 1. Was the outcome determined appropriately? | | | | Y |
|  | 2. Was a prespecified or standard outcome definition used? | | | | Y |
|  | 3. Were predictors excluded from the outcome definition? | | | | Y |
|  | 4. Was the outcome defined and determined in a similar way for all participants? | | | | Y |
|  | 5. Was the outcome determined without knowledge of predictor information? | | | | Y |
|  | 6. Was the time interval between predictor assessment and outcome determination appropriate? | | | | Y |
| Bias rating | *Rationale of bias rating:* | | | | **low** |
| **II. Applicability** | | | | | |
| Concern that included participants or the setting do not match the review question | | Applicability rating | | | **low** |
|  |  | *Rationale of applicability rating:* | | | |
| **Domain 4: Analysis** | | | | | |
| *The support for judgment box：*  Events per variable: 6  Missing data: patients with missing data were excluded.  Modelling method: multivariate logistic regression  Selection of predictors for inclusion: fCP 6 months and all other variables shown to have a significant association with ER in the univariate models.  Model performance: AUC of ROC, sensitivity, specificity (A cut-off maximizes the Youden index) | | | | | |
| **Risk of Bias** | | | | | |
| Signaling questions | 1. Were there a reasonable number of participants with the outcome? | | | | N |
|  | 2. Were continuous and categorical predictors handled appropriately? | | | | Y |
|  | 3. Were all enrolled participants included in the analysis? | | | | N |
|  | 4. Were participants with missing data handled appropriately? | | | | PN |
|  | 5. Was selection of predictors based on univariable analysis avoided? | | | | N |
|  | 6. Were complexities in the data (e.g., censoring, competing risks, sampling of control participants) accounted for appropriately? | | | | PY |
|  | 7. Were relevant model performance measures evaluated appropriately? | | | | N |
|  | 8. Were model overfitting and optimism in model performance accounted for? | | | | N |
|  | 9. Do predictors and their assigned weights in the final model correspond to the results from the reported multivariable analysis? | | | | Y |
| Bias rating | *Rationale of bias rating:*  Events per variable is <10. The study might suffer from a selection bias as participants with missing data were excluded from the analysis. The predictors are selected based on univariable analysis prior to multivariable analysis. Calibration has not been evaluated and no internal validation technique has been performed. | | | | **high** |
| **Overall Assessment of Risk of Bias and Concerns for Applicability** | | | | | |
| Risk of bias | | | **high** | *Summary:*  Small sample size, complete-case analysis, selection of predictors based on univariable analysis, no calibration or internal validation. | |
| Applicability concerns | | | **low** | *Summary:* | |
|  | | | | | |
| 1. **Moret-Tatay I et al, 2021.** **Specific Plasma MicroRNA Signatures in Predicting and Confirming Crohn's Disease Recurrence: Role and Pathogenic Implications.** | | | | | |
| **Domain 1: Participants** | | | | | |
| *The data sources and the criteria for participant selection:*  prospective and consecutive cohort study.  44 patients with CD underwent surgery presented exclusive ileal disease localization (L1) and were therefore eligible for the study. Patients with other disease localization and/or other comorbidities were excluded. Three patients dropped out before completing the follow-up for various reasons (1 patient was diagnosed with lung neoplasia 16 months after surgery, and 2 were lost to follow-up because they stopped attending the clinic during the first year). | | | | | |
| **I. Risk of Bias** | | | | | |
| Signaling questions | 1. Were appropriate data sources used, e.g., cohort, randomized controlled trial, or nested case-control study data? | | | | Y |
|  | 2. Were all inclusions and exclusions of participants appropriate? | | | | PN |
| Bias rating | *Rationale of bias rating:*  Several patients were excluded from the study for various reasons (loss to follow-up, lack of samples available for the epigenetic study, etc.) | | | | **high** |
| **II. Applicability** | | | | | |
| Concern that included participants or the setting do not match the review question | | Applicability rating | | | **low** |
|  |  | *Rationale of applicability rating:* | | | |
| **Domain 2: Predictors** | | | | | |
| *The support for judgment box：*  34 Plasma MicroRNA | | | | | |
| **I. Risk of Bias** | | | | | |
| Signaling questions | 1. Were predictors defined and assessed in a similar way for all participants? | | | | Y |
|  | 2. Were predictor assessments made without knowledge of outcome data? | | | | Y |
|  | 3. Are all predictors available at the time the model is intended to be used? | | | | Y |
| Bias rating | *Rationale of bias rating:* | | | | **low** |
| **II. Applicability** | | | | | |
| Concern that included participants or the setting do not match the review question | | Applicability rating | | | **low** |
|  |  | *Rationale of applicability rating:* | | | |
| **Domain 3: Outcome** | | | | | |
| *The support for judgment box：*  Outcome: morphological recurrence assessed by ileocolonoscopy or MRE within 6-12 months after surgery (Rutgeerts score≥i2b or Sailer score≥MR2) | | | | | |
| **I. Risk of Bias** | | | | | |
| Signaling questions | 1. Was the outcome determined appropriately? | | | | Y |
|  | 2. Was a prespecified or standard outcome definition used? | | | | Y |
|  | 3. Were predictors excluded from the outcome definition? | | | | Y |
|  | 4. Was the outcome defined and determined in a similar way for all participants? | | | | PY |
|  | 5. Was the outcome determined without knowledge of predictor information? | | | | NI |
|  | 6. Was the time interval between predictor assessment and outcome determination appropriate? | | | | Y |
| Bias rating | *Rationale of bias rating:* | | | | **low** |
| **II. Applicability** | | | | | |
| Concern that included participants or the setting do not match the review question | | Applicability rating | | | **low** |
|  |  | *Rationale of applicability rating:* | | | |
| **Domain 4: Analysis** | | | | | |
| *The support for judgment box：*  Events per variable: <10  Modelling method: an elastic net penalized logistic regression model  Selection of predictors for inclusion: We selected 34 miRNAs (after normalization with the mean of Cq values of each sample) for the inferential cohort where the large sample size(32 samples; 16 in NR and 16 in R) was of importance, given it is the most critical step in determining the final predictive models.  Selection of predictors during modelling: an elastic net penalized logistic regression model, which was adjusted to identify the most influential variables  Shrinkage of regression coefficients: penalized with the elastic net algorithm  Model performance: AUC of ROC  Model evaluation: internal validation (bootstrap with 200 replicates) | | | | | |
| **Risk of Bias** | | | | | |
| Signaling questions | 1. Were there a reasonable number of participants with the outcome? | | | | N |
|  | 2. Were continuous and categorical predictors handled appropriately? | | | | Y |
|  | 3. Were all enrolled participants included in the analysis? | | | | Y |
|  | 4. Were participants with missing data handled appropriately? | | | | Y |
|  | 5. Was selection of predictors based on univariable analysis avoided? | | | | Y |
|  | 6. Were complexities in the data (e.g., censoring, competing risks, sampling of control participants) accounted for appropriately? | | | | PY |
|  | 7. Were relevant model performance measures evaluated appropriately? | | | | N |
|  | 8. Were model overfitting and optimism in model performance accounted for? | | | | PY |
|  | 9. Do predictors and their assigned weights in the final model correspond to the results from the reported multivariable analysis? | | | | PY |
| Bias rating | *Rationale of bias rating:*  Events pre variable is <10. No calibration. | | | | **high** |
| **Overall Assessment of Risk of Bias and Concerns for Applicability** | | | | | |
| Risk of bias | | | **high** | *Summary:*  May suffer from selection bias, small sample size, no calibration. | |
| Applicability concerns | | | **low** | *Summary:* | |
|  | | | | | |
| 1. **Kusunoki K et al, 2021.** **The advanced lung cancer inflammation index predicts outcomes in patients with Crohn's disease after surgical resection.** | | | | | |
| **Domain 1: Participants** | | | | | |
| *The data sources and the criteria for participant selection:*  retrospective cohort study, Mie University Hospital (discovery) and Hyogo Medical University Hospital (external validation).  CD patients who underwent intestinal resections were enrolled. Patients who had a follow-up period of less than 5 years after intestinal resection or had insufficient clinical and surgical information were excluded. | | | | | |
| **I. Risk of Bias** | | | | | |
| Signaling questions | 1. Were appropriate data sources used, e.g., cohort, randomized controlled trial, or nested case-control study data? | | | | Y |
|  | 2. Were all inclusions and exclusions of participants appropriate? | | | | PN |
| Bias rating | *Rationale of bias rating:*  Patients who had a follow-up period of less than 5 years after intestinal resection or had insufficient clinical and surgical information were excluded. | | | | **high** |
| **II. Applicability** | | | | | |
| Concern that included participants or the setting do not match the review question | | Applicability rating | | | **low** |
|  |  | *Rationale of applicability rating:* | | | |
| **Domain 2: Predictors** | | | | | |
| *The support for judgment box：*  a nutrition marker: the advanced lung cancer inflammation index (ALI) | | | | | |
| **I. Risk of Bias** | | | | | |
| Signaling questions | 1. Were predictors defined and assessed in a similar way for all participants? | | | | Y |
|  | 2. Were predictor assessments made without knowledge of outcome data? | | | | Y |
|  | 3. Are all predictors available at the time the model is intended to be used? | | | | Y |
| Bias rating | *Rationale of bias rating:* | | | | **low** |
| **II. Applicability** | | | | | |
| Concern that included participants or the setting do not match the review question | | Applicability rating | | | **low** |
|  |  | *Rationale of applicability rating:* | | | |
| **Domain 3: Outcome** | | | | | |
| *The support for judgment box：*  Outcome: Surgical relapse within 5 years (CD-related surgery required due to refractoriness to medical treatments or CD-related complications).  The median follow-up time was 72.5 months [mean ± standard deviation (SD): 71.4 ± 37.8]. | | | | | |
| **I. Risk of Bias** | | | | | |
| Signaling questions | 1. Was the outcome determined appropriately? | | | | Y |
|  | 2. Was a prespecified or standard outcome definition used? | | | | Y |
|  | 3. Were predictors excluded from the outcome definition? | | | | Y |
|  | 4. Was the outcome defined and determined in a similar way for all participants? | | | | Y |
|  | 5. Was the outcome determined without knowledge of predictor information? | | | | NI |
|  | 6. Was the time interval between predictor assessment and outcome determination appropriate? | | | | PY |
| Bias rating | *Rationale of bias rating:* | | | | **low** |
| **II. Applicability** | | | | | |
| Concern that included participants or the setting do not match the review question | | Applicability rating | | | **low** |
|  |  | *Rationale of applicability rating:* | | | |
| **Domain 4: Analysis** | | | | | |
| *The support for judgment box：*  Modelling method: Cox proportional hazards analysis  Selection of predictors for inclusion: After univariate analysis, we selected all variables with P value <0.05 for multivariate analysis based on a Cox proportional hazards regression model.  Model performance: AUC of ROC, sensitivity, specificity  Model evaluation: external validation | | | | | |
| **Risk of Bias** | | | | | |
| Signaling questions | 1. Were there a reasonable number of participants with the outcome? | | | | N |
|  | 2. Were continuous and categorical predictors handled appropriately? | | | | Y |
|  | 3. Were all enrolled participants included in the analysis? | | | | Y |
|  | 4. Were participants with missing data handled appropriately? | | | | Y |
|  | 5. Was selection of predictors based on univariable analysis avoided? | | | | N |
|  | 6. Were complexities in the data (e.g., censoring, competing risks, sampling of control participants) accounted for appropriately? | | | | PY |
|  | 7. Were relevant model performance measures evaluated appropriately? | | | | N |
|  | 8. Were model overfitting and optimism in model performance accounted for? | | | | PY |
|  | 9. Do predictors and their assigned weights in the final model correspond to the results from the reported multivariable analysis? | | | | Y |
| Bias rating | *Rationale of bias rating:*  The number of participants with outcome is small. Variables with P value <0.05 in the univariate analysis were selected for multivariate analysis. No calibration has been evaluated. | | | | **high** |
| **Overall Assessment of Risk of Bias and Concerns for Applicability** | | | | | |
| Risk of bias | | | **high** | *Summary:*  May suffer from selection bias, small sample size, selection of predictors based on univariable analysis, no calibration. | |
| Applicability concerns | | | **low** | *Summary:* | |
|  | | | | | |
| 1. **De Cruz P et al, 2021.** **Endoscopic Prediction of Crohn's Disease Postoperative Recurrence.** | | | | | |
| **Domain 1: Participants** | | | | | |
| *The data sources and the criteria for participant selection:*  part of the POCER study (a prospective randomized controlled trial).  The present subgroup analysis of the POCER study included 85 of the 122 active care study arm patients who underwent postoperative colonoscopy at 6 and 18 months as per protocol and had a Crohn s Disease Activity Index (CDAI) score calculation at both 6 and 18 months postoperatively. | | | | | |
| **I. Risk of Bias** | | | | | |
| Signaling questions | 1. Were appropriate data sources used, e.g., cohort, randomized controlled trial, or nested case-control study data? | | | | Y |
|  | 2. Were all inclusions and exclusions of participants appropriate? | | | | Y |
| Bias rating | *Rationale of bias rating:* | | | | **low** |
| **II. Applicability** | | | | | |
| Concern that included participants or the setting do not match the review question | | Applicability rating | | | **low** |
|  |  | *Rationale of applicability rating:* | | | |
| **Domain 2: Predictors** | | | | | |
| *The support for judgment box：*  6-month postoperative endoscopic parameters: anastomotic ulcer depth (superficial vs deep), number of ulcers (0, ≤2, >2), ulcer size (1-5 mm, ≥6 mm), circumferential extent of ulceration (<25%, ≥25%), and the presence or absence of stenosis | | | | | |
| **I. Risk of Bias** | | | | | |
| Signaling questions | 1. Were predictors defined and assessed in a similar way for all participants? | | | | Y |
|  | 2. Were predictor assessments made without knowledge of outcome data? | | | | Y |
|  | 3. Are all predictors available at the time the model is intended to be used? | | | | Y |
| Bias rating | *Rationale of bias rating:* | | | | **low** |
| **II. Applicability** | | | | | |
| Concern that included participants or the setting do not match the review question | | Applicability rating | | | **low** |
|  |  | *Rationale of applicability rating:* | | | |
| **Domain 3: Outcome** | | | | | |
| *The support for judgment box：*  Outcome: 1. endoscopic recurrence at 18 months (Rutgeert score≥i2); 2. complete mucosal healing (Rutgeerts score = i0) at 18 months. | | | | | |
| **I. Risk of Bias** | | | | | |
| Signaling questions | 1. Was the outcome determined appropriately? | | | | Y |
|  | 2. Was a prespecified or standard outcome definition used? | | | | Y |
|  | 3. Were predictors excluded from the outcome definition? | | | | Y |
|  | 4. Was the outcome defined and determined in a similar way for all participants? | | | | Y |
|  | 5. Was the outcome determined without knowledge of predictor information? | | | | NI |
|  | 6. Was the time interval between predictor assessment and outcome determination appropriate? | | | | Y |
| Bias rating | *Rationale of bias rating:* | | | | **unclear** |
| **II. Applicability** | | | | | |
| Concern that included participants or the setting do not match the review question | | Applicability rating | | | **low** |
|  |  | *Rationale of applicability rating:* | | | |
| **Domain 4: Analysis** | | | | | |
| *The support for judgment box：*  Events per variable: <10  Modelling method: The significance and accuracy of endoscopic lesions identified at 6 months in relation to the subsequent outcome at 18 months were evaluated in isolation and in combination using the stepwise addition of endoscopic factors together with bootstrapping. Based on the stepwise addition of factors and bootstrapping the endoscopic lesions at 6 months, which were associated with outcome at 18 months with the greatest accuracy, were identified and a new endoscopic index of severity was derived.  Model performance: AUC of ROC, sensitivity, specificity, positive predictive value, negative predictive value. Cutoff value was determined using bootstrap logistic regression in combination with the senspec command in Stata 15 and the Youden Index.  Model evaluation: Because of the lack of available validation data, Monte Carlo Markov simulation with 100 repetitions was used to simulate the probabilistic cohort of 200 participants, using priori probabilities derived from the original sample. The model was simulated with 100 repetitions within each simulation, and regression estimates were summarized. | | | | | |
| **Risk of Bias** | | | | | |
| Signaling questions | 1. Were there a reasonable number of participants with the outcome? | | | | N |
|  | 2. Were continuous and categorical predictors handled appropriately? | | | | NI |
|  | 3. Were all enrolled participants included in the analysis? | | | | Y |
|  | 4. Were participants with missing data handled appropriately? | | | | PY |
|  | 5. Was selection of predictors based on univariable analysis avoided? | | | | Y |
|  | 6. Were complexities in the data (e.g., censoring, competing risks, sampling of control participants) accounted for appropriately? | | | | Y |
|  | 7. Were relevant model performance measures evaluated appropriately? | | | | N |
|  | 8. Were model overfitting and optimism in model performance accounted for? | | | | PY |
|  | 9. Do predictors and their assigned weights in the final model correspond to the results from the reported multivariable analysis? | | | | PY |
| Bias rating | *Rationale of bias rating:*  Events per variable is <10. No calibration has been evaluated. | | | | **high** |
| **Overall Assessment of Risk of Bias and Concerns for Applicability** | | | | | |
| Risk of bias | | | **high** | *Summary:*  Small sample size, no calibration. | |
| Applicability concerns | | | **low** | *Summary:* | |
|  | | | | | |
| 1. **Akiyama S et al, 2021.** **Predictability of simple endoscopic score for Crohn's disease for postoperative outcomes in Crohn's disease.** | | | | | |
| **Domain 1: Participants** | | | | | |
| *The data sources and the criteria for participant selection:*  Retrospective study.  This study included CD patients who had a postoperative ileocolonoscopy at the University of Chicago between 2005 and 2016.  We included not only the classical ileocecectomy or ileocolectomy but also other types of intestinal resection. We excluded patients who had already developed the outcome before or at the closest clinical visit to the first ileocolonoscopy after surgery. | | | | | |
| **I. Risk of Bias** | | | | | |
| Signaling questions | 1. Were appropriate data sources used, e.g., cohort, randomized controlled trial, or nested case-control study data? | | | | Y |
|  | 2. Were all inclusions and exclusions of participants appropriate? | | | | Y |
| Bias rating | *Rationale of bias rating:* | | | | **low** |
| **II. Applicability** | | | | | |
| Concern that included participants or the setting do not match the review question | | Applicability rating | | | **low** |
|  |  | *Rationale of applicability rating:* | | | |
| **Domain 2: Predictors** | | | | | |
| *The support for judgment box：*  SES-CD at the first postoperative ileocolonoscopy | | | | | |
| **I. Risk of Bias** | | | | | |
| Signaling questions | 1. Were predictors defined and assessed in a similar way for all participants? | | | | Y |
|  | 2. Were predictor assessments made without knowledge of outcome data? | | | | Y |
|  | 3. Are all predictors available at the time the model is intended to be used? | | | | Y |
| Bias rating | *Rationale of bias rating:* | | | | **low** |
| **II. Applicability** | | | | | |
| Concern that included participants or the setting do not match the review question | | Applicability rating | | | **low** |
|  |  | *Rationale of applicability rating:* | | | |
| **Domain 3: Outcome** | | | | | |
| *The support for judgment box：*  Outcome: clinical recurrence (greater than 4 points of the Harvey–Bradshaw index)  Patients included developed clinical recurrence at a median of 36 months (range 0.6-109 months).  Clinical recurrence of SI-dominant disease, “Small intestine (SI)-dominant disease” was defined as patients without colonic involvement at the first postoperative ileocolonoscopy or patients with Montreal classification L1.  Clinical recurrence of colon-dominant disease, We defined patients with postoperative colonic inflammation at the first ileocolonoscopy after the surgery or patients with Montreal classification L2 as “colon-dominant disease”. | | | | | |
| **I. Risk of Bias** | | | | | |
| Signaling questions | 1. Was the outcome determined appropriately? | | | | Y |
|  | 2. Was a prespecified or standard outcome definition used? | | | | Y |
|  | 3. Were predictors excluded from the outcome definition? | | | | Y |
|  | 4. Was the outcome defined and determined in a similar way for all participants? | | | | Y |
|  | 5. Was the outcome determined without knowledge of predictor information? | | | | NI |
|  | 6. Was the time interval between predictor assessment and outcome determination appropriate? | | | | PY |
| Bias rating | *Rationale of bias rating:* | | | | **low** |
| **II. Applicability** | | | | | |
| Concern that included participants or the setting do not match the review question | | Applicability rating | | |  |
|  |  | *Rationale of applicability rating:* | | | |
| **Domain 4: Analysis** | | | | | |
| *The support for judgment box：*  The number of participants with outcome is small.  Model performance: AUC of ROC, sensitivity, specificity | | | | | |
| **Risk of Bias** | | | | | |
| Signaling questions | 1. Were there a reasonable number of participants with the outcome? | | | | N |
|  | 2. Were continuous and categorical predictors handled appropriately? | | | | Y |
|  | 3. Were all enrolled participants included in the analysis? | | | | Y |
|  | 4. Were participants with missing data handled appropriately? | | | | PY |
|  | 5. Was selection of predictors based on univariable analysis avoided? | | | | Not applicable |
|  | 6. Were complexities in the data (e.g., censoring, competing risks, sampling of control participants) accounted for appropriately? | | | | PN |
|  | 7. Were relevant model performance measures evaluated appropriately? | | | | N |
|  | 8. Were model overfitting and optimism in model performance accounted for? | | | | Not applicable |
|  | 9. Do predictors and their assigned weights in the final model correspond to the results from the reported multivariable analysis? | | | | Not applicable |
| Bias rating | *Rationale of bias rating:*  The number of participants with outcome is small. The possibility that the postoperative outcomes could be affected by treatments after surgery has not been excluded. No calibration has been evaluated. | | | | **high** |
| **Overall Assessment of Risk of Bias and Concerns for Applicability** | | | | | |
| Risk of bias | | | **high** | *Summary:*  Small sample size, no calibration, no treatment information. | |
| Applicability concerns | | | **low** | *Summary:* | |
|  | | | | | |
| 1. **Sokol H et al, 2020.** **Prominence of ileal mucosa-associated microbiota to predict postoperative endoscopic recurrence in Crohn's disease.** | | | | | |
| **Domain 1: Participants** | | | | | |
| *The data sources and the criteria for participant selection:*  a large prospective multicentric cohort study.  Inclusion criteria were age >18 years, ileal or ileocolonic CD, and an indication of CD related intestinal surgery (ileocolonic resection) in the absence of intestinal dysplasia or cancer.  Patients who receive antibiotics in the last month before surgery were excluded from the analysis. | | | | | |
| **I. Risk of Bias** | | | | | |
| Signaling questions | 1. Were appropriate data sources used, e.g., cohort, randomized controlled trial, or nested case-control study data? | | | | Y |
|  | 2. Were all inclusions and exclusions of participants appropriate? | | | | Y |
| Bias rating | *Rationale of bias rating:* | | | | **low** |
| **II. Applicability** | | | | | |
| Concern that included participants or the setting do not match the review question | | Applicability rating | | | **low** |
|  |  | *Rationale of applicability rating:* | | | |
| **Domain 2: Predictors** | | | | | |
| *The support for judgment box：*  9 gut microbiota taxa from the surgical specimen in the inflamed portion (M0) of the ileum and 3 clinical factors [male gender, active smoking, and previous intestinal resection] | | | | | |
| **I. Risk of Bias** | | | | | |
| Signaling questions | 1. Were predictors defined and assessed in a similar way for all participants? | | | | Y |
|  | 2. Were predictor assessments made without knowledge of outcome data? | | | | Y |
|  | 3. Are all predictors available at the time the model is intended to be used? | | | | Y |
| Bias rating | *Rationale of bias rating:* | | | | **low** |
| **II. Applicability** | | | | | |
| Concern that included participants or the setting do not match the review question | | Applicability rating | | | **low** |
|  |  | *Rationale of applicability rating:* | | | |
| **Domain 3: Outcome** | | | | | |
| *The support for judgment box：*  Outcome: Postoperative recurrence about 6-12 months after surgery (Rutgeerts score ≥ i2) | | | | | |
| **I. Risk of Bias** | | | | | |
| Signaling questions | 1. Was the outcome determined appropriately? | | | | Y |
|  | 2. Was a prespecified or standard outcome definition used? | | | | Y |
|  | 3. Were predictors excluded from the outcome definition? | | | | Y |
|  | 4. Was the outcome defined and determined in a similar way for all participants? | | | | Y |
|  | 5. Was the outcome determined without knowledge of predictor information? | | | | NI |
|  | 6. Was the time interval between predictor assessment and outcome determination appropriate? | | | | Y |
| Bias rating | *Rationale of bias rating:* | | | | **unclear** |
| **II. Applicability** | | | | | |
| Concern that included participants or the setting do not match the review question | | Applicability rating | | | **low** |
|  |  | *Rationale of applicability rating:* | | | |
| **Domain 4: Analysis** | | | | | |
| *The support for judgment box：*  Events per variable: <10  Modelling method: random forest  Model performance: AUC of ROC  Model evaluation: the population of interest was split in a training set (70% of the population of interest, randomly chosen) and a validation set (remaining 30% of the population) | | | | | |
| **Risk of Bias** | | | | | |
| Signaling questions | 1. Were there a reasonable number of participants with the outcome? | | | | N |
|  | 2. Were continuous and categorical predictors handled appropriately? | | | | Y |
|  | 3. Were all enrolled participants included in the analysis? | | | | N |
|  | 4. Were participants with missing data handled appropriately? | | | | NI |
|  | 5. Was selection of predictors based on univariable analysis avoided? | | | | Y |
|  | 6. Were complexities in the data (e.g., censoring, competing risks, sampling of control participants) accounted for appropriately? | | | | PY |
|  | 7. Were relevant model performance measures evaluated appropriately? | | | | PN |
|  | 8. Were model overfitting and optimism in model performance accounted for? | | | | Y |
|  | 9. Do predictors and their assigned weights in the final model correspond to the results from the reported multivariable analysis? | | | | Y |
| Bias rating | *Rationale of bias rating:*  Events per variable is <10. Patients who received postoperative anti-TNF agents were not included in the analysis. Calibration has not been evaluated. | | | | **high** |
| **Overall Assessment of Risk of Bias and Concerns for Applicability** | | | | | |
| Risk of bias | | | **high** | *Summary:*  Small sample size, lack of information about missing data, no calibration. | |
| Applicability concerns | | | **low** | *Summary:* | |
|  | | | | | |
| 1. **Machiels K et al, 2020.** **Early Postoperative Endoscopic Recurrence in Crohn's Disease Is Characterised by Distinct Microbiota Recolonisation.** | | | | | |
| **Domain 1: Participants** | | | | | |
| *The data sources and the criteria for participant selection:*  prospective cohort, University Hospitals Leuven [Belgium]  121 patients with CD who underwent an ileocaecal resection between 2011 and 2016 | | | | | |
| **I. Risk of Bias** | | | | | |
| Signaling questions | 1. Were appropriate data sources used, e.g., cohort, randomized controlled trial, or nested case-control study data? | | | | Y |
|  | 2. Were all inclusions and exclusions of participants appropriate? | | | | PY |
| Bias rating | *Rationale of bias rating:* | | | | **low** |
| **II. Applicability** | | | | | |
| Concern that included participants or the setting do not match the review question | | Applicability rating | | | **low** |
|  |  | *Rationale of applicability rating:* | | | |
| **Domain 2: Predictors** | | | | | |
| *The support for judgment box：*  microbial factors [fecal and mucosal], clinical factors at the time of surgery | | | | | |
| **I. Risk of Bias** | | | | | |
| Signaling questions | 1. Were predictors defined and assessed in a similar way for all participants? | | | | Y |
|  | 2. Were predictor assessments made without knowledge of outcome data? | | | | Y |
|  | 3. Are all predictors available at the time the model is intended to be used? | | | | Y |
| Bias rating | *Rationale of bias rating:* | | | | **low** |
| **II. Applicability** | | | | | |
| Concern that included participants or the setting do not match the review question | | Applicability rating | | | **low** |
|  |  | *Rationale of applicability rating:* | | | |
| **Domain 3: Outcome** | | | | | |
| *The support for judgment box：*  Outcome: 6-month postoperative recurrence [POR] (Rutgeerts score ≥i2b) | | | | | |
| **I. Risk of Bias** | | | | | |
| Signaling questions | 1. Was the outcome determined appropriately? | | | | Y |
|  | 2. Was a prespecified or standard outcome definition used? | | | | Y |
|  | 3. Were predictors excluded from the outcome definition? | | | | Y |
|  | 4. Was the outcome defined and determined in a similar way for all participants? | | | | Y |
|  | 5. Was the outcome determined without knowledge of predictor information? | | | | NI |
|  | 6. Was the time interval between predictor assessment and outcome determination appropriate? | | | | Y |
| Bias rating | *Rationale of bias rating:* | | | | **unclear** |
| **II. Applicability** | | | | | |
| Concern that included participants or the setting do not match the review question | | Applicability rating | | | **low** |
|  |  | *Rationale of applicability rating:* | | | |
| **Domain 4: Analysis** | | | | | |
| *The support for judgment box：*  Events per variable: <10  Modelling method: Decision tree (C5.0 algorithm)  Model performance: AUC of ROC  Model evaluation: internal validation (Random Forest algorithm) | | | | | |
| **Risk of Bias** | | | | | |
| Signaling questions | 1. Were there a reasonable number of participants with the outcome? | | | | N |
|  | 2. Were continuous and categorical predictors handled appropriately? | | | | Y |
|  | 3. Were all enrolled participants included in the analysis? | | | | Y |
|  | 4. Were participants with missing data handled appropriately? | | | | NI |
|  | 5. Was selection of predictors based on univariable analysis avoided? | | | | Y |
|  | 6. Were complexities in the data (e.g., censoring, competing risks, sampling of control participants) accounted for appropriately? | | | | PY |
|  | 7. Were relevant model performance measures evaluated appropriately? | | | | PY |
|  | 8. Were model overfitting and optimism in model performance accounted for? | | | | Y |
|  | 9. Do predictors and their assigned weights in the final model correspond to the results from the reported multivariable analysis? | | | | Y |
| Bias rating | *Rationale of bias rating:*  The number of participants with the outcome is relatively small. | | | | **high** |
| **Overall Assessment of Risk of Bias and Concerns for Applicability** | | | | | |
| Risk of bias | | | **high** | *Summary:*  Small sample size, lack of information about missing data. | |
| Applicability concerns | | | **low** | *Summary:* | |
|  | | | | | |
| 1. **Ikeda A et al, 2019.** **A Novel Predictive Nomogram for Early Endoscopic Recurrence after Intestinal Resection for Crohn's Disease.** | | | | | |
| **Domain 1: Participants** | | | | | |
| *The data sources and the criteria for participant selection:*  single-center, retrospective study.  1) included data on consecutive patients with CD who received intestinal resections for macroscopic disease at Osaka University between April 2008 and 2017.  2) Patients were excluded when they had no endoscopic evaluation between 6 and 12 months after surgery; when they received an intestinal resection with a diverting ileostomy or did not receive an anastomosis; when their follow-up data were lost; or when detailed data were lacking. | | | | | |
| **I. Risk of Bias** | | | | | |
| Signaling questions | 1. Were appropriate data sources used, e.g., cohort, randomized controlled trial, or nested case-control study data? | | | | Y |
|  | 2. Were all inclusions and exclusions of participants appropriate? | | | | PN |
| Bias rating | *Rationale of bias rating:*  Patients were excluded when detailed data were lacking. | | | | **high** |
| **II. Applicability** | | | | | |
| Concern that included participants or the setting do not match the review question | | Applicability rating | | | **low** |
|  |  | *Rationale of applicability rating:* | | | |
| **Domain 2: Predictors** | | | | | |
| *The support for judgment box：*  baseline characteristics, perioperative medications, surgery, and perioperative laboratory findings | | | | | |
| **I. Risk of Bias** | | | | | |
| Signaling questions | 1. Were predictors defined and assessed in a similar way for all participants? | | | | Y |
|  | 2. Were predictor assessments made without knowledge of outcome data? | | | | NI |
|  | 3. Are all predictors available at the time the model is intended to be used? | | | | Y |
| Bias rating | *Rationale of bias rating:*  Use of objective predictors not requiring subjective interpretation. | | | | **low** |
| **II. Applicability** | | | | | |
| Concern that included participants or the setting do not match the review question | | Applicability rating | | | **low** |
|  |  | *Rationale of applicability rating:* | | | |
| **Domain 3: Outcome** | | | | | |
| *The support for judgment box：*  Outcome: 6-12 months postoperative endoscopic recurrence (Rutgeert score≥i2b) | | | | | |
| **I. Risk of Bias** | | | | | |
| Signaling questions | 1. Was the outcome determined appropriately? | | | | Y |
|  | 2. Was a prespecified or standard outcome definition used? | | | | Y |
|  | 3. Were predictors excluded from the outcome definition? | | | | Y |
|  | 4. Was the outcome defined and determined in a similar way for all participants? | | | | Y |
|  | 5. Was the outcome determined without knowledge of predictor information? | | | | NI |
|  | 6. Was the time interval between predictor assessment and outcome determination appropriate? | | | | Y |
| Bias rating | *Rationale of bias rating:* | | | | **unclear** |
| **II. Applicability** | | | | | |
| Concern that included participants or the setting do not match the review question | | Applicability rating | | | **low** |
|  |  | *Rationale of applicability rating:* | | | |
| **Domain 4: Analysis** | | | | | |
| *The support for judgment box：*  Events per variable: <10  Modelling method: Logistic regression  Selection of predictors for inclusion: based on the risk factors identified in the univariate analyses  Model performance: AUC of ROC  Model evaluation: It was validated with an internal validation method, based on the same study patients used to develop the model. | | | | | |
| **Risk of Bias** | | | | | |
| Signaling questions | 1. Were there a reasonable number of participants with the outcome? | | | | N |
|  | 2. Were continuous and categorical predictors handled appropriately? | | | | PY |
|  | 3. Were all enrolled participants included in the analysis? | | | | Y |
|  | 4. Were participants with missing data handled appropriately? | | | | Y |
|  | 5. Was selection of predictors based on univariable analysis avoided? | | | | N |
|  | 6. Were complexities in the data (e.g., censoring, competing risks, sampling of control participants) accounted for appropriately? | | | | PY |
|  | 7. Were relevant model performance measures evaluated appropriately? | | | | N |
|  | 8. Were model overfitting and optimism in model performance accounted for? | | | | NI |
|  | 9. Do predictors and their assigned weights in the final model correspond to the results from the reported multivariable analysis? | | | | Y |
| Bias rating | *Rationale of bias rating:*  Events per variable is <10. Risk factors identified in the univariate analyses were included for multivariable modeling. Calibration has not been evaluated. No information is provided on how to apply internal validation. | | | | **high** |
| **Overall Assessment of Risk of Bias and Concerns for Applicability** | | | | | |
| Risk of bias | | | **high** | *Summary:*  May suffer from selection bias, small sample size, selection of predictor based on univariable analysis, no calibration and lack of detailed information about internal validation. | |
| Applicability concerns | | | **low** | *Summary:* | |
|  | | | | | |
| 1. **Cushing KC et al, 2019.** **Predicting Risk of Postoperative Disease Recurrence in Crohn's Disease: Patients With Indolent Crohn's Disease Have Distinct Whole Transcriptome Profiles at the Time of First Surgery.** | | | | | |
| **Domain 1: Participants** | | | | | |
| *The data sources and the criteria for participant selection:*  electronic medical record.  Inclusion criteria were a diagnosis of CD, first ileocolic resection [ICR], interval colonoscopy, and whole transcriptome array meeting quality control standards. | | | | | |
| **I. Risk of Bias** | | | | | |
| Signaling questions | 1. Were appropriate data sources used, e.g., cohort, randomized controlled trial, or nested case-control study data? | | | | Y |
|  | 2. Were all inclusions and exclusions of participants appropriate? | | | | Y |
| Bias rating | *Rationale of bias rating:* | | | | **low** |
| **II. Applicability** | | | | | |
| Concern that included participants or the setting do not match the review question | | Applicability rating | | | **low** |
|  |  | *Rationale of applicability rating:* | | | |
| **Domain 2: Predictors** | | | | | |
| *The support for judgment box：*  transcripts from mucosal biopsies of uninflamed tissue from operative specimens | | | | | |
| **I. Risk of Bias** | | | | | |
| Signaling questions | 1. Were predictors defined and assessed in a similar way for all participants? | | | | Y |
|  | 2. Were predictor assessments made without knowledge of outcome data? | | | | NI |
|  | 3. Are all predictors available at the time the model is intended to be used? | | | | Y |
| Bias rating | *Rationale of bias rating:*  Use of objective predictors not requiring subjective interpretation. | | | | **low** |
| **II. Applicability** | | | | | |
| Concern that included participants or the setting do not match the review question | | Applicability rating | | | **low** |
|  |  | *Rationale of applicability rating:* | | | |
| **Domain 3: Outcome** | | | | | |
| *The support for judgment box：*  Outcome: 1) TNF-Naïve Cohort: differential classification of Rutgeerts score i0 vs i1-i4 of the first postoperative endoscopy [i0: complete mucosal remission]; 2) TNF-Exposed Cohort: differential classification of aggressive (a composite score≥14) and indolent (score≤8) disease.  Medical treatment and timing of endoscopic follow-up were heterogeneous. | | | | | |
| **I. Risk of Bias** | | | | | |
| Signaling questions | 1. Was the outcome determined appropriately? | | | | Y |
|  | 2. Was a prespecified or standard outcome definition used? | | | | N |
|  | 3. Were predictors excluded from the outcome definition? | | | | Y |
|  | 4. Was the outcome defined and determined in a similar way for all participants? | | | | Y |
|  | 5. Was the outcome determined without knowledge of predictor information? | | | | NI |
|  | 6. Was the time interval between predictor assessment and outcome determination appropriate? | | | | PN |
| Bias rating | *Rationale of bias rating:*  The timing of endoscopic follow-up was heterogeneous. The definition of outcome was not generally accepted. | | | | **high** |
| **II. Applicability** | | | | | |
| Concern that included participants or the setting do not match the review question | | Applicability rating | | | **low** |
|  |  | *Rationale of applicability rating:* | | | |
| **Domain 4: Analysis** | | | | | |
| *The support for judgment box：*  Events per variable: <10  Missing data: patients with missing data were excluded from analysis  Modelling method: Random Forest, classification  Selection of predictors during modelling: mean decrease accuracy; mean decrease Gini  Model performance: an out-of-bag estimate of error rate | | | | | |
| **Risk of Bias** | | | | | |
| Signaling questions | 1. Were there a reasonable number of participants with the outcome? | | | | N |
|  | 2. Were continuous and categorical predictors handled appropriately? | | | | Y |
|  | 3. Were all enrolled participants included in the analysis? | | | | N |
|  | 4. Were participants with missing data handled appropriately? | | | | Y |
|  | 5. Was selection of predictors based on univariable analysis avoided? | | | | Y |
|  | 6. Were complexities in the data (e.g., censoring, competing risks, sampling of control participants) accounted for appropriately? | | | | NI |
|  | 7. Were relevant model performance measures evaluated appropriately? | | | | N |
|  | 8. Were model overfitting and optimism in model performance accounted for? | | | | N |
|  | 9. Do predictors and their assigned weights in the final model correspond to the results from the reported multivariable analysis? | | | | Y |
| Bias rating | *Rationale of bias rating:*  Events per variable is <10. Five patients were excluded from analysis due to extreme variability on whole transcriptome analysis. Calibration has not been evaluated. No internal or external validation. | | | | **high** |
| **Overall Assessment of Risk of Bias and Concerns for Applicability** | | | | | |
| Risk of bias | | | **high** | *Summary:*  Heterogeneous follow-up time, small sample size, no calibration or validation. The definition of outcome was not generally accepted. | |
| Applicability concerns | | | **low** | *Summary:* | |
|  | | | | | |
| 1. **Cerrillo E et al, 2019.** **A Nomogram Combining Fecal Calprotectin Levels and Plasma Cytokine Profiles for Individual Prediction of Postoperative Crohn s Disease Recurrence.** | | | | | |
| **Domain 1: Participants** | | | | | |
| *The data sources and the criteria for participant selection:*  prospective, single-center study, tertiary referral center.  All consecutive patients who had undergone ileocecal or ileocolonic resection for active CD (including previous anastomosis) were prospectively included over a period of 4 years (from 2011 to 2014). All the patients were followed up for 24 months after surgery (or until evidence of morphological recurrence appeared). | | | | | |
| **I. Risk of Bias** | | | | | |
| Signaling questions | 1. Were appropriate data sources used, e.g., cohort, randomized controlled trial, or nested case-control study data? | | | | Y |
|  | 2. Were all inclusions and exclusions of participants appropriate? | | | | Y |
| Bias rating | *Rationale of bias rating:* | | | | low |
| **II. Applicability** | | | | | |
| Concern that included participants or the setting do not match the review question | | Applicability rating | | | **low** |
|  |  | *Rationale of applicability rating:* | | | |
| **Domain 2: Predictors** | | | | | |
| *The support for judgment box：*  fecal, clinical, demographic, serological variables (FC at 6 month, IL-13 presurgery, IL-6 and IFN-γ at 6 months) | | | | | |
| **I. Risk of Bias** | | | | | |
| Signaling questions | 1. Were predictors defined and assessed in a similar way for all participants? | | | | Y |
|  | 2. Were predictor assessments made without knowledge of outcome data? | | | | Y |
|  | 3. Are all predictors available at the time the model is intended to be used? | | | | Y |
| Bias rating | *Rationale of bias rating:* | | | | **low** |
| **II. Applicability** | | | | | |
| Concern that included participants or the setting do not match the review question | | Applicability rating | | | **low** |
|  |  | *Rationale of applicability rating:* | | | |
| **Domain 3: Outcome** | | | | | |
| *The support for judgment box：*  Outcome: morphological recurrence was assessed by ileocolonoscopy or magnetic resonance enterography within 6-12 months after surgery: 1. Endoscopic recurrence (Rutgeert score≥i2b); 2. Radiological recurrence (Sailer index≥MR2). Blind assessment. | | | | | |
| **I. Risk of Bias** | | | | | |
| Signaling questions | 1. Was the outcome determined appropriately? | | | | Y |
|  | 2. Was a prespecified or standard outcome definition used? | | | | Y |
|  | 3. Were predictors excluded from the outcome definition? | | | | Y |
|  | 4. Was the outcome defined and determined in a similar way for all participants? | | | | Y |
|  | 5. Was the outcome determined without knowledge of predictor information? | | | | Y |
|  | 6. Was the time interval between predictor assessment and outcome determination appropriate? | | | | Y |
| Bias rating | *Rationale of bias rating:* | | | | **low** |
| **II. Applicability** | | | | | |
| Concern that included participants or the setting do not match the review question | | Applicability rating | | | **low** |
|  |  | *Rationale of applicability rating:* | | | |
| **Domain 4: Analysis** | | | | | |
| *The support for judgment box：*  Events per variable: <10  Modelling method: Logistic regression  Model performance: AUC of ROC | | | | | |
| **Risk of Bias** | | | | | |
| Signaling questions | 1. Were there a reasonable number of participants with the outcome? | | | | N |
|  | 2. Were continuous and categorical predictors handled appropriately? | | | | PY |
|  | 3. Were all enrolled participants included in the analysis? | | | | N |
|  | 4. Were participants with missing data handled appropriately? | | | | Y |
|  | 5. Was selection of predictors based on univariable analysis avoided? | | | | Y |
|  | 6. Were complexities in the data (e.g., censoring, competing risks, sampling of control participants) accounted for appropriately? | | | | PY |
|  | 7. Were relevant model performance measures evaluated appropriately? | | | | N |
|  | 8. Were model overfitting and optimism in model performance accounted for? | | | | N |
|  | 9. Do predictors and their assigned weights in the final model correspond to the results from the reported multivariable analysis? | | | | Y |
| Bias rating | *Rationale of bias rating:*  Events per variable is <10. Six patients dropped out before completing follow-up for various reasons. Calibration and internal validation have not been performed. | | | | **high** |
| **Overall Assessment of Risk of Bias and Concerns for Applicability** | | | | | |
| Risk of bias | | | **high** | *Summary:*  Small sample size, no calibration or internal validation. Patients dropped out before completing follow-up were exclude from analysis. | |
| Applicability concerns | | | **low** | *Summary:* | |
|  | | | | | |
| 1. **Auzoux J et al, 2019.** **Usefulness of confocal laser endomicroscopy for predicting postoperative recurrence in patients with Crohn's disease: a pilot study.** | | | | | |
| **Domain 1: Participants** | | | | | |
| *The data sources and the criteria for participant selection:*  prospective cohort study.  1) From October 2010 to December 2013, 25 consecutive CD patients, aged >18 years, who had an ileocolonic resection performed in the previous 6 to 12 months and who underwent a first routine ileocolonoscopy with ileal intubation were prospectively enrolled in the study at Lyon-Sud University Hospital.  2) All patients had a curative ileocolonic resection with complete resection of macroscopically involved bowel for ileocolonic or terminal ileal CD. On surgical specimen examination, the margins were free of any inflammation.  3) Patients aged <18 years, pregnant women, patients with stomas, and patients with impaired renal function or known allergy to fluorescein were excluded from the study.  4) Only patients in endoscopic remission (Rutgeerts <i2) at the first ileocolonoscopy were followed-up until clinical relapse or an endoscopic recurrence. | | | | | |
| **I. Risk of Bias** | | | | | |
| Signaling questions | 1. Were appropriate data sources used, e.g., cohort, randomized controlled trial, or nested case-control study data? | | | | Y |
|  | 2. Were all inclusions and exclusions of participants appropriate? | | | | Y |
| Bias rating | *Rationale of bias rating:* | | | | **low** |
| **II. Applicability** | | | | | |
| Concern that included participants or the setting do not match the review question | | Applicability rating | | | **low** |
|  |  | *Rationale of applicability rating:* | | | |
| **Domain 2: Predictors** | | | | | |
| *The support for judgment box：*  a score for Confocal laser endomicroscopy [CLE] images: Watson score | | | | | |
| **I. Risk of Bias** | | | | | |
| Signaling questions | 1. Were predictors defined and assessed in a similar way for all participants? | | | | Y |
|  | 2. Were predictor assessments made without knowledge of outcome data? | | | | Y |
|  | 3. Are all predictors available at the time the model is intended to be used? | | | | Y |
| Bias rating | *Rationale of bias rating:* | | | | **low** |
| **II. Applicability** | | | | | |
| Concern that included participants or the setting do not match the review question | | Applicability rating | | | **low** |
|  |  | *Rationale of applicability rating:* | | | |
| **Domain 3: Outcome** | | | | | |
| *The support for judgment box：*  The median follow-up was 38 months. Outcome:  1) endoscopic recurrence (Rutgeerts≥i2)  2) clinical relapse [the occurrence or increase of symptoms associated with a Harvey-Bradshaw index score >5 at 2 consecutive examinations separated by 7 days and an elevated fecal calprotectin result (>250 mg/g stool)] | | | | | |
| **I. Risk of Bias** | | | | | |
| Signaling questions | 1. Was the outcome determined appropriately? | | | | Y |
|  | 2. Was a prespecified or standard outcome definition used? | | | | Y |
|  | 3. Were predictors excluded from the outcome definition? | | | | PY |
|  | 4. Was the outcome defined and determined in a similar way for all participants? | | | | Y |
|  | 5. Was the outcome determined without knowledge of predictor information? | | | | NI |
|  | 6. Was the time interval between predictor assessment and outcome determination appropriate? | | | | PY |
| Bias rating | *Rationale of bias rating:* | | | | **unclear** |
| **II. Applicability** | | | | | |
| Concern that included participants or the setting do not match the review question | | Applicability rating | | | **low** |
|  |  | *Rationale of applicability rating:* | | | |
| **Domain 4: Analysis** | | | | | |
| *The support for judgment box：*  Events per variable: <10  Model performance: AUC of ROC, sensitivity, specificity, positive predictive value, negative predictive value, overall accuracy | | | | | |
| **Risk of Bias** | | | | | |
| Signaling questions | 1. Were there a reasonable number of participants with the outcome? | | | | N |
|  | 2. Were continuous and categorical predictors handled appropriately? | | | | Y |
|  | 3. Were all enrolled participants included in the analysis? | | | | PY |
|  | 4. Were participants with missing data handled appropriately? | | | | NI |
|  | 5. Was selection of predictors based on univariable analysis avoided? | | | | Not applicable |
|  | 6. Were complexities in the data (e.g., censoring, competing risks, sampling of control participants) accounted for appropriately? | | | | PY |
|  | 7. Were relevant model performance measures evaluated appropriately? | | | | N |
|  | 8. Were model overfitting and optimism in model performance accounted for? | | | | Not applicable |
|  | 9. Do predictors and their assigned weights in the final model correspond to the results from the reported multivariable analysis? | | | | Not applicable |
| Bias rating | *Rationale of bias rating:*  The number of participants with the outcome is small. No calibration. | | | | **high** |
| **Overall Assessment of Risk of Bias and Concerns for Applicability** | | | | | |
| Risk of bias | | | **high** | *Summary:*  Small sample size, lack of information about missing data, no calibration. | |
| Applicability concerns | | | **low** | *Summary:* | |
|  | | | | | |
| 1. **Nakao S et al, 2017.** **Predictive value of myenteric and submucosal plexitis for postoperative Crohn's disease recurrence.** | | | | | |
| **Domain 1: Participants** | | | | | |
| *The data sources and the criteria for participant selection:*  retrospective study of a single-center cohort study.  Patients with ileocolonic or colonic resection and ileocolonic or colo-colonic anastomosis, and who were over 20 years of age at the time of the study, were included.  Exclusion criteria were absence of a labeled proximal margin and unavailability for postoperative follow-up for more than 2 years. | | | | | |
| **I. Risk of Bias** | | | | | |
| Signaling questions | 1. Were appropriate data sources used, e.g., cohort, randomized controlled trial, or nested case-control study data? | | | | Y |
|  | 2. Were all inclusions and exclusions of participants appropriate? | | | | PY |
| Bias rating | *Rationale of bias rating:* | | | | **low** |
| **II. Applicability** | | | | | |
| Concern that included participants or the setting do not match the review question | | Applicability rating | | | **low** |
|  |  | *Rationale of applicability rating:* | | | |
| **Domain 2: Predictors** | | | | | |
| *The support for judgment box：*  clinical and pathological [resection specimens] variables | | | | | |
| **I. Risk of Bias** | | | | | |
| Signaling questions | 1. Were predictors defined and assessed in a similar way for all participants? | | | | Y |
|  | 2. Were predictor assessments made without knowledge of outcome data? | | | | Y |
|  | 3. Are all predictors available at the time the model is intended to be used? | | | | Y |
| Bias rating | *Rationale of bias rating:* | | | | **low** |
| **II. Applicability** | | | | | |
| Concern that included participants or the setting do not match the review question | | Applicability rating | | | **low** |
|  |  | *Rationale of applicability rating:* | | | |
| **Domain 3: Outcome** | | | | | |
| *The support for judgment box：*  Outcome: Significant endoscopic recurrence was defined as Rutgeerts score of i2 or higher confined to the anastomotic site (within 2.5 centimeters from the anastomosis to both oral and anal side).  The mean duration from surgery to endoscopic investigation was 33.6±31.9 months.  The mean interval between surgery and endoscopic recurrence was 49.7±34.7 months. | | | | | |
| **I. Risk of Bias** | | | | | |
| Signaling questions | 1. Was the outcome determined appropriately? | | | | Y |
|  | 2. Was a prespecified or standard outcome definition used? | | | | Y |
|  | 3. Were predictors excluded from the outcome definition? | | | | Y |
|  | 4. Was the outcome defined and determined in a similar way for all participants? | | | | Y |
|  | 5. Was the outcome determined without knowledge of predictor information? | | | | NI |
|  | 6. Was the time interval between predictor assessment and outcome determination appropriate? | | | | PY |
| Bias rating | *Rationale of bias rating:* | | | | **unclear** |
| **II. Applicability** | | | | | |
| Concern that included participants or the setting do not match the review question | | Applicability rating | | | **low** |
|  |  | *Rationale of applicability rating:* | | | |
| **Domain 4: Analysis** | | | | | |
| *The support for judgment box：*  Events per variable: <10  Missing data: patients with missing data were excluded from analysis  Modelling method: multiple logistic regression  Selection of predictors for inclusion: all significant variables evaluated on univariate analysis were integrated into multiple logistic regression  Model performance: R-squared, AUC of ROC | | | | | |
| **Risk of Bias** | | | | | |
| Signaling questions | 1. Were there a reasonable number of participants with the outcome? | | | | N |
|  | 2. Were continuous and categorical predictors handled appropriately? | | | | PY |
|  | 3. Were all enrolled participants included in the analysis? | | | | N |
|  | 4. Were participants with missing data handled appropriately? | | | | N |
|  | 5. Was selection of predictors based on univariable analysis avoided? | | | | N |
|  | 6. Were complexities in the data (e.g., censoring, competing risks, sampling of control participants) accounted for appropriately? | | | | NI |
|  | 7. Were relevant model performance measures evaluated appropriately? | | | | N |
|  | 8. Were model overfitting and optimism in model performance accounted for? | | | | N |
|  | 9. Do predictors and their assigned weights in the final model correspond to the results from the reported multivariable analysis? | | | | Y |
| Bias rating | *Rationale of bias rating:*  Events per variable is <10. Patients with missing data were excluded from analysis. All significant variables evaluated on univariate analysis were integrated into multiple logistic regression. Calibration and internal validation have not been performed. | | | | **high** |
| **Overall Assessment of Risk of Bias and Concerns for Applicability** | | | | | |
| Risk of bias | | | **high** | *Summary:*  Small sample size, complete-case analysis, selection of predictor based on univariate analysis, no calibration or validation. | |
| Applicability concerns | | | **low** | *Summary:* | |
|  | | | | | |
| 1. **Hamilton AL et al, 2017.** **Serologic antibodies in relation to outcome in postoperative Crohn's disease.** | | | | | |
| **Domain 1: Participants** | | | | | |
| *The data sources and the criteria for participant selection:*  prospective, randomized, multi-center trial.  1. Inclusion Criteria: 1) clinical diagnosis of Crohn's and proven history of disease; 2) patient undergone surgical resection with creation of a primary anastomosis.  2. Exclusion Criteria: 1) endoscopically inaccessible anastomosis by standard colonoscopy; 2) presence of an end stoma; 3) pregnancy; 4) suspected perforation of the gastrointestinal tract; 5) presence of certain unsuitable comorbidities. | | | | | |
| **I. Risk of Bias** | | | | | |
| Signaling questions | 1. Were appropriate data sources used, e.g., cohort, randomized controlled trial, or nested case-control study data? | | | | Y |
|  | 2. Were all inclusions and exclusions of participants appropriate? | | | | Y |
| Bias rating | *Rationale of bias rating:* | | | | **low** |
| **II. Applicability** | | | | | |
| Concern that included participants or the setting do not match the review question | | Applicability rating | | | **low** |
|  |  | *Rationale of applicability rating:* | | | |
| **Domain 2: Predictors** | | | | | |
| *The support for judgment box：*  eight serological antibodies | | | | | |
| **I. Risk of Bias** | | | | | |
| Signaling questions | 1. Were predictors defined and assessed in a similar way for all participants? | | | | Y |
|  | 2. Were predictor assessments made without knowledge of outcome data? | | | | Y |
|  | 3. Are all predictors available at the time the model is intended to be used? | | | | Y |
| Bias rating | *Rationale of bias rating:* | | | | **low** |
| **II. Applicability** | | | | | |
| Concern that included participants or the setting do not match the review question | | Applicability rating | | | **low** |
|  |  | *Rationale of applicability rating:* | | | |
| **Domain 3: Outcome** | | | | | |
| *The support for judgment box：*  Outcome: 6 or 18 months postoperative endoscopic recurrence (Rutgeert score≥i2) | | | | | |
| **I. Risk of Bias** | | | | | |
| Signaling questions | 1. Was the outcome determined appropriately? | | | | Y |
|  | 2. Was a prespecified or standard outcome definition used? | | | | Y |
|  | 3. Were predictors excluded from the outcome definition? | | | | Y |
|  | 4. Was the outcome defined and determined in a similar way for all participants? | | | | Y |
|  | 5. Was the outcome determined without knowledge of predictor information? | | | | NI |
|  | 6. Was the time interval between predictor assessment and outcome determination appropriate? | | | | Y |
| Bias rating | *Rationale of bias rating:* | | | | **unclear** |
| **II. Applicability** | | | | | |
| Concern that included participants or the setting do not match the review question | | Applicability rating | | | **low** |
|  |  | *Rationale of applicability rating:* | | | |
| **Domain 4: Analysis** | | | | | |
| *The support for judgment box：*  Missing data: patients with missing data were excluded from analysis  Modelling method: quartile sum score; number of positive markers  Model performance: AUC of ROC | | | | | |
| **Risk of Bias** | | | | | |
| Signaling questions | 1. Were there a reasonable number of participants with the outcome? | | | | NI |
|  | 2. Were continuous and categorical predictors handled appropriately? | | | | Y |
|  | 3. Were all enrolled participants included in the analysis? | | | | N |
|  | 4. Were participants with missing data handled appropriately? | | | | N |
|  | 5. Was selection of predictors based on univariable analysis avoided? | | | | Y |
|  | 6. Were complexities in the data (e.g., censoring, competing risks, sampling of control participants) accounted for appropriately? | | | | PY |
|  | 7. Were relevant model performance measures evaluated appropriately? | | | | N |
|  | 8. Were model overfitting and optimism in model performance accounted for? | | | | N |
|  | 9. Do predictors and their assigned weights in the final model correspond to the results from the reported multivariable analysis? | | | | Y |
| Bias rating | *Rationale of bias rating:*  Number of outcomes has not been reported. Patients with missing data were excluded from analysis. Calibration and internal validation have not been performed. | | | | **high** |
| **Overall Assessment of Risk of Bias and Concerns for Applicability** | | | | | |
| Risk of bias | | | **high** | *Summary:*  Lack of number of outcomes, complete-case analysis, no calibration or internal validation. | |
| Applicability concerns | | | **low** | *Summary:* | |

# Supplementary Table 3. Assessment of risk of bias and applicability based on PROBAST

| Author (year) | Risk of bias | | | |  | Applicability | | |  | Overall | |
| --- | --- | --- | --- | --- | --- | --- | --- | --- | --- | --- | --- |
|  | Participants | Predictors | Outcome | Analysis |  | Participants | Predictors | Outcome |  | Risk of bias | Applicability |
| Wu EH (2022)^18^ | **−** | **+** | **?** | **−** |  | **+** | **+** | **+** |  | **−** | **+** |
| Wang MH (2021)^19^ | **+** | **+** | **?** | **−** |  | **+** | **+** | **+** |  | **−** | **+** |
| Primas C (2021)^12^ | **+** | **+** | **+** | **−** |  | **+** | **+** | **+** |  | **−** | **+** |
| Moret-Tatay I (2021)^13^ | **−** | **+** | **+** | **−** |  | **+** | **+** | **+** |  | **−** | **+** |
| Kusunoki K (2021)^20^ | **−** | **+** | **+** | **−** |  | **+** | **+** | **+** |  | **−** | **+** |
| De Cruz P (2021)^25^ | **+** | **+** | **?** | **−** |  | **+** | **+** | **+** |  | **−** | **+** |
| Akiyama S (2021)^21^ | **+** | **+** | **+** | **−** |  | **+** | **+** | **+** |  | **−** | **+** |
| Sokol H (2020)^14^ | **+** | **+** | **?** | **−** |  | **+** | **+** | **+** |  | **−** | **+** |
| Machiels K (2020)^15^ | **+** | **+** | **?** | **−** |  | **+** | **+** | **+** |  | **−** | **+** |
| Ikeda A (2019)^22^ | **−** | **+** | **?** | **−** |  | **+** | **+** | **+** |  | **−** | **+** |
| Cushing KC (2019)^23^ | **+** | **+** | **−** | **−** |  | **+** | **+** | **+** |  | **−** | **+** |
| Cerrillo E (2019)^16^ | **+** | **+** | **+** | **−** |  | **+** | **+** | **+** |  | **−** | **+** |
| Auzoux J (2019)^17^ | **+** | **+** | **?** | **−** |  | **+** | **+** | **+** |  | **−** | **+** |
| Nakao S (2017)^24^ | **+** | **+** | **?** | **−** |  | **+** | **+** | **+** |  | **−** | **+** |
| Hamilton AL (2017)^26^ | **+** | **+** | **?** | **−** |  | **+** | **+** | **+** |  | **−** | **+** |

+: low risk of bias/ applicability concern; −: high risk of bias/applicability concern; ?: unclear risk of bias/applicability concern.

# Supplementary Table 4. The main predictors for postoperative recurrence

| Types of predictors | Endoscopic recurrence | Surgical recurrence | Clinical recurrence |
| --- | --- | --- | --- |
| Clinical factors | Male gender  Age at diagnosis  **Active smoking***^a^*  Perforating disease  Initial intestinal resection  **Previous intestinal resection***^a^*  Postoperative prophylactic treatment | Body mass index  Penetrating behaviour  Upper gastrointestinal disease  Emergency at initial surgery  Staged surgery at initial surgery  Early era of first intestinal surgery  Immunomodulatory use |  |
| Endoscopic or pathological variables | Circumferential involvement  Deep ulceration  Functional mucosal defect with fluorescein leakage  Functional and structural mucosal defects with micro-erosions and fluorescein leakage  Rate of plexitis  Size of the most severely inflamed ganglion in the myenteric plexus  Total number of inflammatory cells in the submucosal plexus |  | Ulcerated surfaces  Affected surfaces  Ulcer size  Stenosis |
| Serological variables | Albumin  Excessive perioperative inflammation (preoperative CRP+ POD 7 CRP + a peak postoperative CRP)  Interferon-γ  Interleukin-6  Anti-*Saccharomyces cerevisiae* antibodies IgA and IgG  Anti-CBir1 IgG  Anti-OmpC IgA  Anti-A4-Fla2 IgG  Anti-Fla-X IgG | Albumin  Neutrophil to Lymphocyte Ratio |  |
| Fecal variables | **faecal calprotectin***^a^* |  |  |
| Genetic  variables | rs2060886  hsa-miR-93-5p  hsa-miR-191-5p  hsa-miR-451a  hsa-miR-15b-5p  hsa-miR-106b-5p |  |  |
| Microbiota | *Gammaproteobacteria*  *R. gnavus group.Other Streptococcus*  *Ralstonia*  *Haemophilus*  *Gemella*  *Phasolarctobacterium*  *Coprobacilus*  Unidentified *Lachnospiraceae* genus  *Dorea* |  |  |

*^a^*These predictors were mentioned more than one times.

CRP, C-reactive protein; POD, postoperative day.
